# Heme-substituted protein assembly bridged by synthetic porphyrin: achieving controlled configuration while maintaining rotational freedom†

**DOI:** 10.1039/d4ra01042f

**Published:** 2024-03-15

**Authors:** Hiroaki Inaba, Yuma Shisaka, Shinya Ariyasu, Erika Sakakibara, Garyo Ueda, Yuichiro Aiba, Nobutaka Shimizu, Hiroshi Sugimoto, Osami Shoji

**Affiliations:** a Department of Chemistry, School of Science, Nagoya University Furo-cho, Chikusa-ku Nagoya Aichi 464-0802 Japan shoji.osami.w3@f.mail.nagoya-u.ac.jp; b Photon Factory, Institute of Materials Structure Science, High Energy Accelerator Research Organization (KEK) 1-1 Oho Tsukuba Ibaraki 305-0801 Japan; c RIKEN SPring-8 Center 1-1-1 Kouto Sayo Hyogo 679-5148 Japan

## Abstract

The use of biological host–guest interactions, specifically the binding of hemoprotein to heme, has attracted significant research interest in the design of artificial protein assemblies. However, because of the inherent flexibility of the propionic acid group of heme, it is difficult to control the positioning and orientation of the protein unit and to construct well-ordered structures. Herein, we report a heme-substituted protein dimer composed of the native hemoprotein HasA, which accommodates a tetraphenylporphyrin bearing an additional metal coordination site. The specific binding of the tetraphenylporphyrin with an additional metal coordination site that protrudes in a fixed direction confines the configuration of the dimer structure to a defined bent form. The small-angle X-ray scattering profile shows the dimer structure with a bent form and suggests dynamic rotational behavior while keeping its bent-core structure, resembling a bevel gear. This unique dimer structure demonstrates that the design of heme-substituted protein assemblies can be expanded to protein assemblies while maintaining the rotational freedom of the individual protein units.

## Abstract 2

Construction of a bevel-gear-like protein assembly using a synthetic porphyrin with defined flexibility.

## Graphical abstract

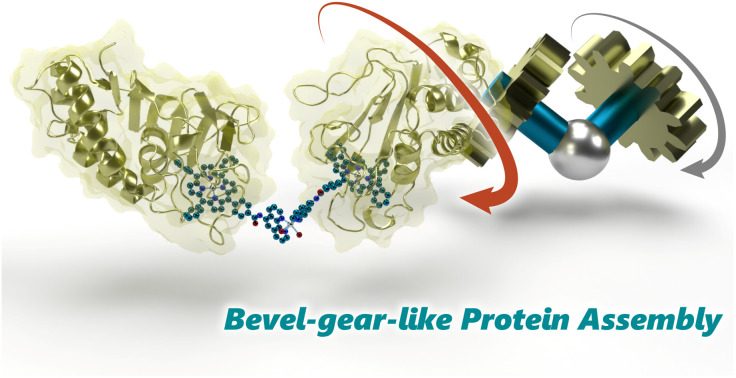

## Introduction

Protein self-assembly plays a pivotal role in expressing the diverse range of functions of proteins in nature. Recent decades have seen the development of a wide array of artificial protein assemblies that mimic the self-assembly of natural proteins.^1^ Artificial protein assemblies have the potential to serve as protein-based materials with functions that emulate or exceed those of natural protein assemblies.^1^ Various strategies have been developed to induce and control self-assembly through metal coordination,^2–5^ covalent bonding,^6–8^ host–guest interactions,^9–15^ and so on. Using biological host–guest interactions, such as hemoprotein–heme,^9–11^ avidin–biotin,^12,13^ and lectin–carbohydrate^14,15^ is one of the promising approaches to the design of artificial protein assemblies. Among the artificial protein assemblies reported, the use of hemoproteins has garnered significant research interest because the propionic acid of heme can be readily modified, and the modified heme can be placed specifically at the heme-binding site in heme proteins. By incorporating a modified heme bearing additional interacting sites to induce protein assembly, we can create artificial protein assemblies based on the interaction of the modified heme; therefore, it is possible to control assemblies by changing the modifier.^9–11^ However, because of the inherent flexibility of the propionic acid group of heme, it is difficult to control the positioning and orientation of the protein unit and construct well-ordered structures. If it were possible to incorporate a synthetic metal complex, in which additional interacting sites such as metal ligands are connected to the porphyrin ring by a rigid linker, into hemoproteins, it would enable the construction of protein assemblies with controlled protein-to-protein arrangement and orientation. However, it is generally challenging to achieve the stable incorporation of metal complexes with structures different from heme into hemoproteins.

**Fig. 1:**
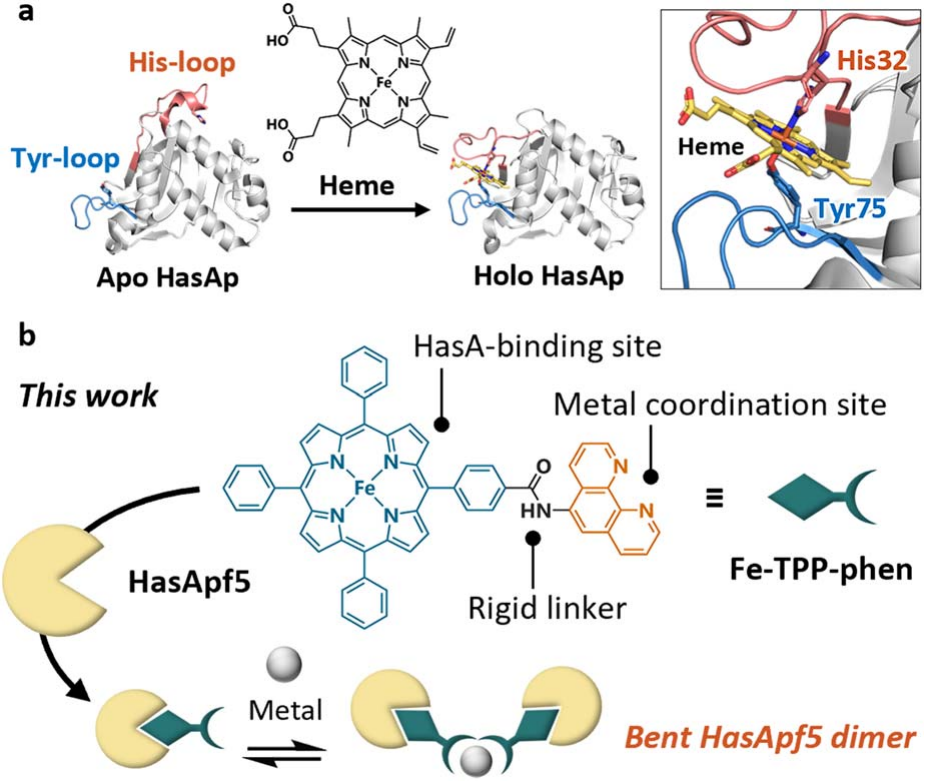
(a) Heme acquisition system protein A (HasA). The crystal structures of the heme-free form (apo HasAp, PDB ID: 3MOK), and the heme-bound form (holo HasAp, PDB ID: 3ELL) show significant conformational changes upon heme-binding. (b) Schematic view of dimerized HasA using a TPP derivative bearing an additional metal coordination site protruding in a fixed direction.

Recently, we have successfully expanded the scope of available synthetic metal complexes for heme substitution in the heme acquisition system protein A (HasAp)^16–21^ which is a hemophore secreted by *Pseudomonas aeruginosa* under iron-deficient conditions.^22–25^ The crystal structure of the heme-binding form of HasA (Fig. 1a) shows that the heme is coordinated by His32 and Tyr75. These residues, which are situated in two separate loops, hold the heme molecule securely in a manner reminiscent of a pair of tweezers, with the captured heme highly exposed to the solvent.^24,25^ Notably, the loop containing His32 undergoes a significant conformational change upon binding with heme.^25^ Based on these HasA characteristics, we expected that HasAp could stably incorporate a plethora of synthetic metal complexes with structures that differ greatly from heme, and we succeeded in incorporating several synthetic metal complexes including iron(iii)-tetraphenylporphyrin (Fe-TPP).^16–21^

TPP has a highly symmetric and rigid skeleton, and a variety of TPP derivatives can be produced by using established synthetic methods.^26–29^ These characteristics have been used in the design of synthetic supermolecules such as metal–organic frameworks^30,31^ and caged metal complexes,^32,33^ where they contribute to the control of the assembled structures. Considering the design flexibility of TPP, resulting from its established synthetic methods, and the potential of the derivatives to be incorporated in HasAp, we expected that well-defined protein assemblies could be prepared by incorporating TPP derivatives bearing a metal coordination site into HasA.

Herein we report the dimerization of HasApf5 derived from *Pseudomonas protegens* PF-5,^34^ by incorporating Fe-TPP with a phenanthroline (phen) ligand as an additional metal coordination site (Fig. 1b). By the addition of Ni^2+^ ions, HasApf5 binds Fe-TPP-phen to form a stable dimer structure with a bent configuration. Interestingly, the HasApf5 exhibits dynamic rotation behavior while keeping the bent-core structure.

## Results and discussion

**Fig. 2:**
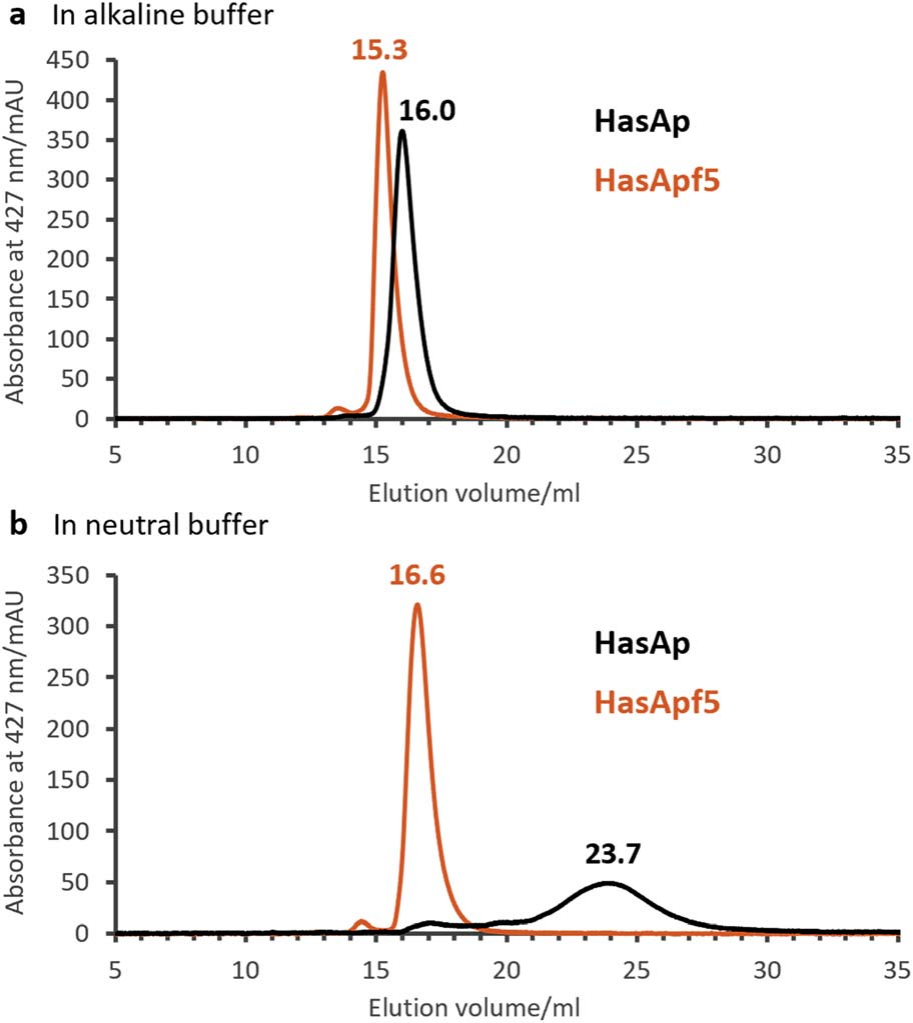
Size-exclusion chromatography (SEC) analysis of HasAp (black) and HasApf5 (red) capturing Fe-TPP detecting Soret band (427 nm). (a) Fe-TPP HasAs in 0.1 M CHES–KOH pH 9.5. (b) Fe-TPP HasAs in 0.1 M KPi pH 7.0.

To explore the types of metal complexes that could be incorporated into HasA originated from bacteria having the heme acquisition system, we found that HasApf5, derived from *Pseudomonas protegens* PF-5, can form a more stable complex with Fe-TPP than HasAp. Size-exclusion chromatography (SEC) analysis of HasApf5 bound to Fe-TPP showed a single sharp peak under both alkaline and neutral conditions (pH 7.0–9.5), whereas Fe-TPP bound to HasAp gave a single sharp peak only under alkaline conditions (Fig. 2a and b). The UV-vis spectra of HasApf5 bound to Fe-TPP recorded after several days were almost identical to the initial spectra even under neutral conditions (Fig. S2†), suggesting that the Fe-TPP binding is highly stable. Furthermore, HasApf5 capture of Fe-TPP was stable even at high temperatures. The *T*_m_ value of HasApf5 capture of Fe-TPP was estimated to be 60.5 °C, which is relatively high and comparable to that of HasAp-bound Fe-TPP under alkaline conditions (62.4 °C) (Table S1†).

**Fig. 3:**
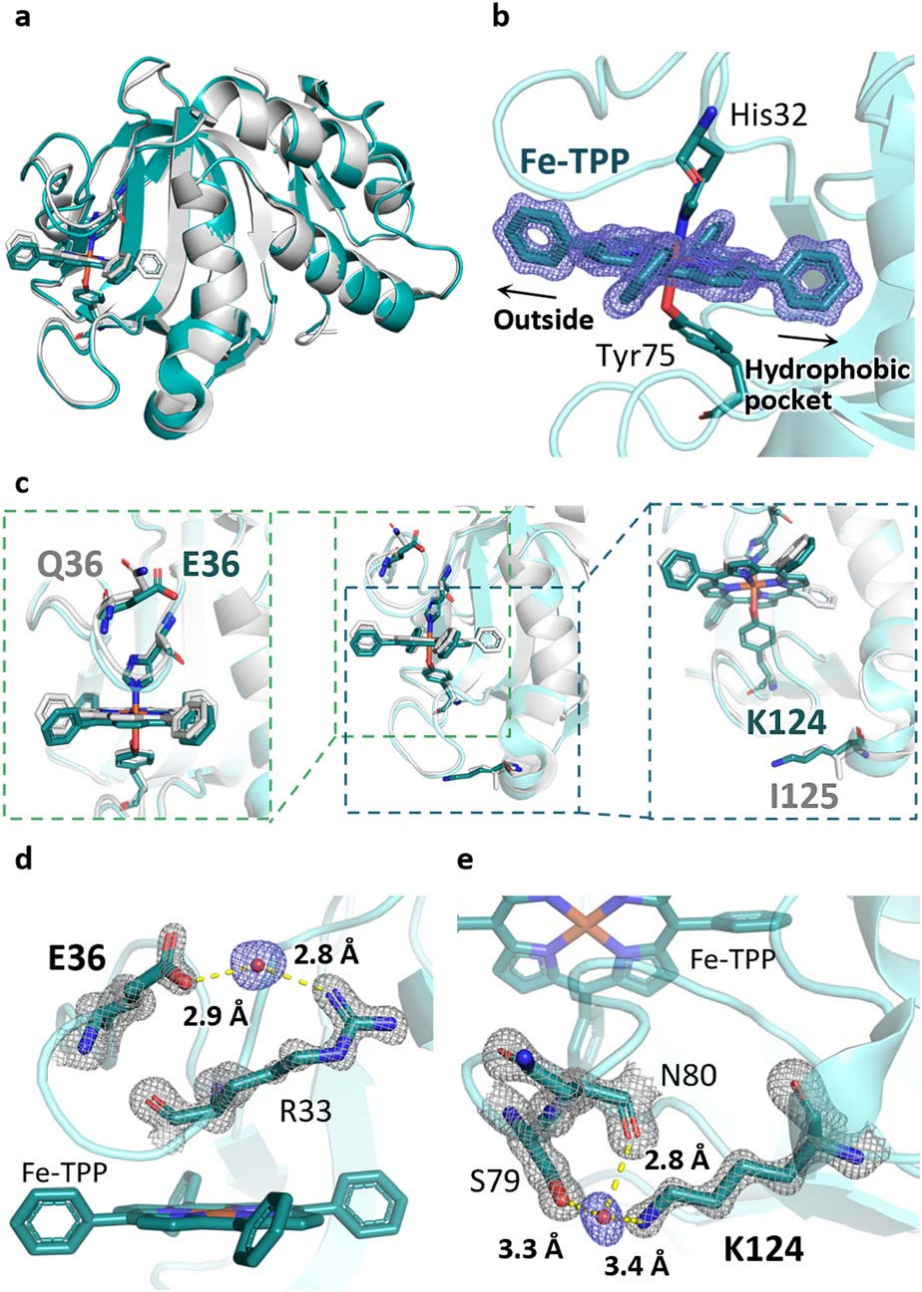
(a) Superimposed image of Fe-TPP HasApf5 (PDB ID: 8KI0, green) and Fe-TPP HasAp (PDB ID: 7EMO, gray). (b) Fe-TPP-binding site of HasApf5. Polder map omitting Fe-TPP contoured at 5.0*σ* (blue mesh). (c) Superimposed image of HasApf5 (green) and HasAp (gray) shows differences in the composition of amino acids in the Fe-TPP-binding site. (d and e) Additional hydrogen bonds mediated by water molecules making up the Fe-TPP-binding site. The hydrogen bonds are shown as dashed lines. 2*F*_o_ − *F*_c_ map contoured at 2.0*σ* (gray mesh). Polder map omitting water molecules contoured at 5.0*σ* (blue mesh).

The crystal structure of HasApf5 with Fe-TPP (Fig. 3a–e) was determined at 1.45 Å resolution. Its overall structure is similar to that of HasAp with Fe-TPP (root-mean-square deviation [RMSD] over amino acid residues 2–180 for Cα atoms: 0.397), and Fe-TPP is positioned in the heme-binding site with coordination by His32 and Tyr75 (Fig. 2a and b). Upon closer examination and comparison, additional hydrogen bonds mediated by water molecules were observed at the Fe-TPP binding site: namely, Glu36-water-Arg33, Lys124-water-Ser79 and -Asn80 (Fig. 3c–e). These additional hydrogen-bonding interactions appear to contribute to the stabilization of the Fe-TPP binding. We would like to mention here that, although not directly related to this report, we have also confirmed the stable incorporation of other metal complexes with HasApf5 such as iron salophen (Fe-salophen), and iron phthalocyanine (Fe-Pc) (Fig. S3†). We believe that the stabilization of HasApf5 with other metal complexes can also be attributed to additional hydrogen-bonding interactions, as confirmed in the crystal structure, which shows that they play a significant role.

**Fig. 4:**
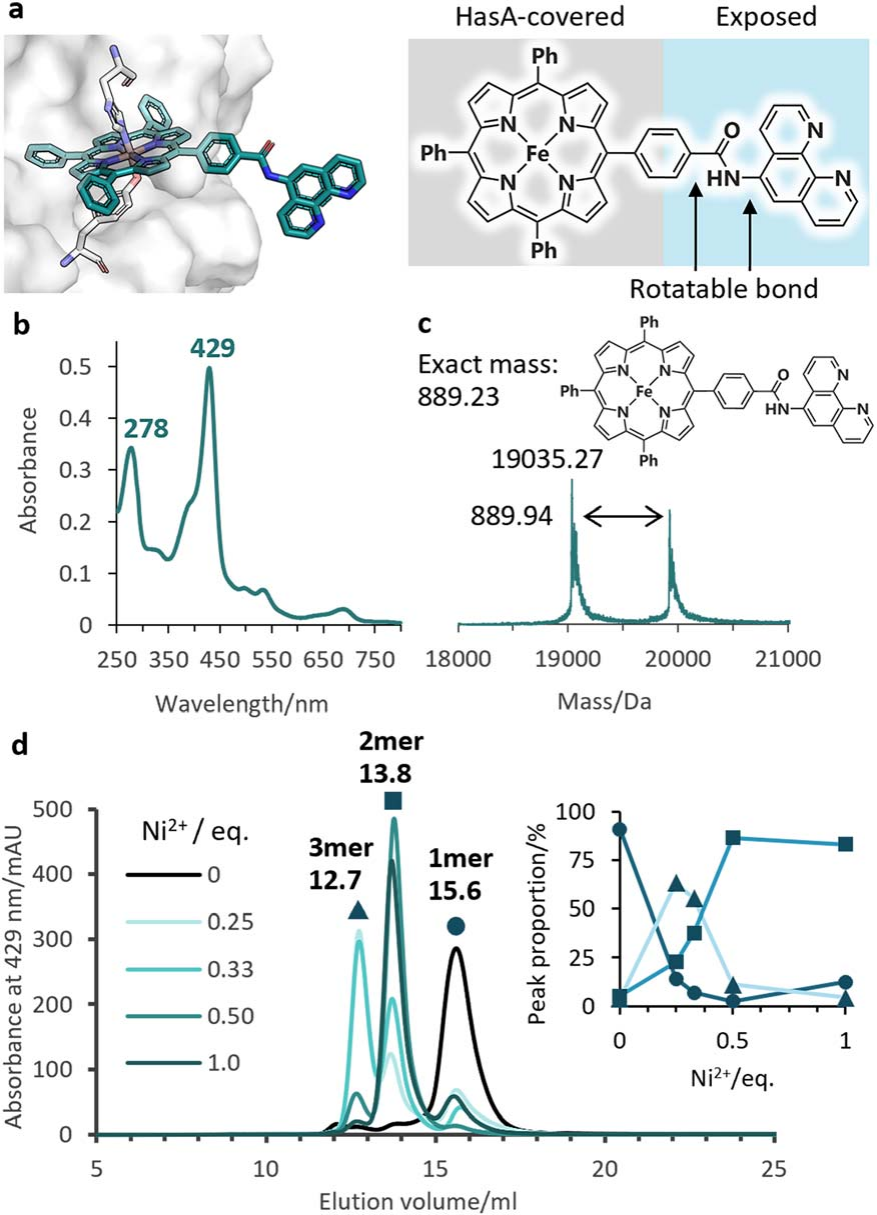
(a) The model structure of HasApf5 with captured Fe-TPP-phen shows the exposed phen ligand. (b) UV-vis spectrum of Fe-TPP-phen HasApf5 in 50 mM CHES–KOH pH 9.5. (c) Positive-mode ESI-TOF mass spectrum of Fe-TPP-phen HasApf5 in 5 mM ammonium acetate buffer. Calculated molecular weight of complex-free HasA (apo HasApf5): 19 034.49. (d) SEC analysis of Fe-TPP-phen HasApf5 (black), with Ni^2+^ (green) in 50 mM CHES–KOH pH 9.5. Peak proportions for the monomer (15.6 mL, circle), dimer (13.8 mL, square), and trimer (12.7 mL, triangle) are shown in the inset.

Given the stable incorporation of Fe-TPP by HasApf5, we then prepared a Fe-TPP derivative, Fe-TPP-phen, bearing a phen ligand linked to one of the phenyl groups of Fe-TPP *via* a planar amide linkage (Fig. 1b). The result of docking simulations of Fe- TPP-phen into HasApf5 using the crystal structure of the Fe-TPP-binding form (Fig. 4a) indicated that the phen ligand is anchored away from the HasApf5 surface, suggesting that the formation of HasApf5 assemblies would be possible through the addition of metal ions. HasApf5 bound to Fe-TPP-phen was prepared by using a reported method^21^ and the UV-vis spectrum and ESI-TOF mass spectrum data confirmed the incorporation of Fe-TPP-phen into HasApf5 (Fig. 4b and c).

We then investigated the multimerization of HasApf5 through metal coordination upon the addition of metal ions (Fe^2+^, Cu^2+^, and Ni^2+^) (Fig. S4†). Among the systems examined, the inclusion of Ni^2+^ ions induced clear peak shifts in the SEC profiles. The stoichiometric ratio of Ni^2+^ ions to phen ligands influenced the proportion of observed peaks corresponding to the monomer, dimer, and trimer of Fe-TPP-phen HasApf5 (Fig. 4d and S5†). The individual peak components were isolated, and, upon isolation, their UV-vis spectra were found to be identical to that of the monomer, which suggests that the coordination environment of the Fe-TPP moiety was preserved (Fig. S6†). As a control experiment, HasApf5 with Fe-TPP was treated with Ni^2+^ ions but no peak shift was observed in the SEC profile (Fig. S7†). As confirmed by SEC measurements, the isolated dimer remained stable in solution for 24 hours within the pH range of 7.0 to 9.5 (Fig. S8 and S9†).

**Fig. 5:**
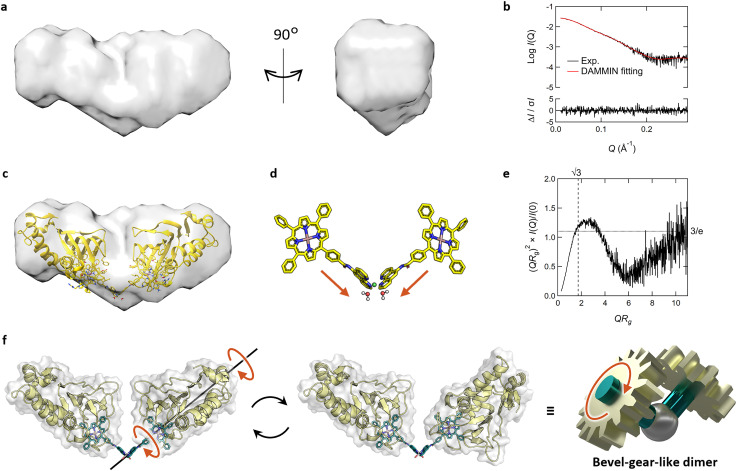
(a–e) SEC-SAXS results of Fe-TPP-phen HasApf5 after Ni^2+^ treatment. (a) Surface density map of the resulting dimer derived from its bead model. (b) The theoretical SAXS profile (red) calculated from the bead model is fitted to the experimental SAXS profile (black). The residuals between both profiles are shown at the bottom of the graph. (c) Superimposed image of the bead model and the predicted structure of *Λ-trans*1 which shows the lowest *χ*^2^ value. (d) The structure of Fe-TPP-phen-Ni complex in the model structure of *Λ-trans*1. (e) Dimensionless Kratky plot of the resulting dimer. (f) Schematic view of bevel-gear-like rotational motion describing the motion of the dimer. (f) Schematic view of bevel-gear-like rotational motion describing the motion of the dimer.

To obtain insights into the dimeric structure in solution, we performed structural analysis by small-angle X-ray scattering analysis combined with SEC (SEC-SAXS) (Fig. S10 and Table S2†). The molecular weight, estimated from *I*(0), aligned precisely with that of the dimer of Fe-TPP-phen HasApf5 (Table S2†). The bead model illustrating the approximate shape of the dimer revealed a bent structure (Fig. 5a and b). To examine possible bent forms of the dimer, we modeled six dimer structures in both bent and liner forms, taking the isomers of the Ni-phen complex moiety into consideration (Fig. S11†). Comparing the experimental profile and the theoretical profile of the six potential structures based on the scores of iterative closest point (ICP) superimpositions^35^ and the *χ*^2^ values (Fig. S12, 13 and Table S2†), all four structures with a bent form gave low *χ*^2^ values (*χ*^2^ = 1.03–1.81), indicating the plausibility of the bent structure (Fig. 5c and d). In contrast, the two structures with linear forms gave high *χ*^2^ values (*χ*^2^ = 5.22–6.09), which also inferred that the dimer structure had a bent form. When the SAXS profile was examined in detail, it was noted that the peak in the dimensionless Kratky plot of the dimer (Fig. 5e) shifts outward and elevates beyond what is observed in a compact, spherical conformation. The profile also exhibits an upward trend in the region *QR*_g_ > 6. This unique characteristic suggests that the dimer has a core structure but with partially unfixed structural regions.

The molecular structure of Fe-TPP-phen shows that two bonds besides the amide bond are rotatable (Fig. 1b and 4a). In fact, the energy diagram of the dihedral rotation between the amide group and the phenyl group of the Fe-TPP moiety within the potential dimer structure with the lowest *χ*^2^ value suggests that this bond is freely rotatable even in the dimeric structure (Δ*E* < 5 kcal mol^−1^) (Fig. S14†). The results of the structural analysis led us to conclude that the resulting dimer exhibits dynamic rotational behavior along the bond rotation while maintaining its bent-core structure; a description that resembles that of a bevel gear (Fig. 5f). Thus, in our molecular design of HasApf5 with an incorporated metal complex, although the binding direction is fixed, the inclusion of a rotationally flexible linkage around the main axis can impart motion to the assembled protein structure. The introduction of defined flexibility into artificial protein assemblies could allow control of their motion, thereby providing them with motion-based functions, leading to the development of dynamic artificial protein assemblies, a field that currently has only limited examples.^7,36,37^

## Conclusions

We constructed a heme-substituted protein assembly using a TPP derivative bearing an additional metal coordination site. This achievement expands the range of designs of heme-substituted protein assemblies, thereby promoting the diversification of assembly structures. The resulting dimer forms a bent structure due to the specific binding of Fe-TPP-phen in which the phen group protrudes in a fixed direction. The dimeric bent structure could serve as a building unit for defined architectures, such as rings, spirals, and cages. In addition, the dynamic bevel-gear-like motion may facilitate the development of protein assemblies akin to molecular machines and enable the expression of functions inherent to such assemblies. Several studies in these directions are ongoing in our research group.

## Experimental

### Preparation of HasA with Fe-TPP

HasA (HasAp or HasApf5) with Fe-TPP was prepared according to the published procedures.^21^ The TPP ligand and its iron complex (Fe-TPP Cl) were prepared according to a method described in the literature.^21,27^ Fe-TPP Cl was solubilized in DMSO, and then mixed with apo HasA in PBS. Following overnight dialysis of a mixture of Fe-TPP/HasA in 50 mM Tris–HCl (pH 8.5), excess Fe-TPP was removed by filtration through a PVDF membrane (0.22 μm, Millipore). The filtrate was loaded onto a Q Sepharose Fast Flow column (GE Healthcare, Cytiva) preequilibrated in 50 mM Tris–HCl (pH 8.5) buffer and washed with at least 3 column volumes of buffer A [0.1 M CHES–KOH (pH 9.5)]. Bound protein was eluted in buffer A containing 0.5 M NaCl. After desalting, the eluates were purified *via* anion exchange chromatography (HiPrep capto DEAE 5 mL; GE Healthcare, Cytiva) to eliminate apo HasA. The loaded sample was washed with 1 column volume of buffer A including 10% buffer B [buffer A with 0.8 M NaCl], and then gradually eluted over 20 column 3 volumes with the ratio of buffer B increasing gradually from 10% to 80%. Purified Fe-TPP HasA was stored in 0.1 M CHES–KOH (pH 9.5) at 4 °C or −80 °C. The final concentration of HasA with Fe-TPP was determined by absorption of the Soret band (Fe-TPP HasAp: *ε*_428_ = 91.9 mM^−1^ cm^−1^,^21^ Fe-TPP HasApf5: *ε*_428_ = 73.8 mM^−1^ cm^−1^ estimated *via* a Pierce™ BCA Protein Assay Kit (Thermo Scientific) using HasAp as the protein standard).

### Preparation of HasApf5 with Fe-TPP-phen

Synthesized Fe-TPP-phen (the synthetic method is described in ESI†) was solubilized in DMSO, and then mixed with apo HasApf5 in PBS. Following overnight dialysis of a mixture of Fe-TPP-phen/HasApf5 in 50 mM Tris–HCl (pH 8.5) with 10 mM EDTA, excess Fe-TPP-phen was removed by filtration through a PVDF membrane (0.22 μm, Millipore). The filtrate was purified *via* size exclusion chromatography (HiPrep 16/60 Sephacryl S-200 HR; GE Healthcare, Cytiva) equilibrated with 50 mM CHES–KOH (pH 9.5). The eluates were further purified *via* immobilized Cu^2+^ affinity chromatography (HiPrep Chelating HP 5 mL; GE Healthcare, Cytiva) to eliminate apo HasApf5. The loaded sample was washed with 1 column volume of buffer A including 0.5% buffer B [50 mM CHES–KOH, 0.5 M imidazole (pH 9.5)], and then gradually eluted over 5 column volumes with the ratio of buffer B increasing gradually from 1% to 10%. After buffer exchange to 50 mM CHES–KOH using a size exclusion chromatography (HiPrep 16/60 Sephacryl S-200 HR; GE Healthcare, Cytiva), purified Fe-TPP-phen HasA was stored at 4 °C. The final protein concentration was estimated *via* a Pierce™ BCA Protein Assay Kit (Thermo Scientific) using HasApf5 as the protein standard. The estimated molar extinction coefficients are as follows: *ε*_429_ = 108.2 mM^−1^ cm^−1^, *ε*_280_ = 68.8 mM^−1^ cm^−1^.

### Measurement

UV-vis spectra were recorded on a UV-2600 PC spectrophotometer (Shimadzu). ESI-TOF mass spectra were recorded on a micrOTOFII (Bruker Daltonics) using positive mode ESI-TOF method for protein solutions.

### Analysis of stability of HasAs capturing Fe-TPP *via* size-exclusion chromatography (SEC)

The stability of HasAs capturing Fe-TPP was evaluated *via* SEC using a Superdex 200 increase 10/300 GL column (GE Healthcare, Cytiva). An 80 μL of Fe-TPP HasA solution (400 μM) was loaded onto the column equilibrated with 0.1 M KPi (pH 7.0) or 0.1 M CHES–KOH (pH 9.5) and then eluted at the flow rate of 0.5 mL min^−1^.

### Crystallization of HasA with Fe-TPP

Purified HasApf5 with Fe-TPP was exchanged into a crystallization buffer [0.1 M CHES–KOH buffer (pH 9.5)] *via* a PD-10 desalting column (GE Healthcare, Cytiva). The eluate was concentrated *via* an Amicon Ultra filter 3 kDa cutoff (Merck Millipore). The final concentration was determined by UV-vis spectroscopy. The crystallization condition was obtained from screening tests using Wizard Classic 1 and 2 (Rigaku). Crystals were grown at 20 °C using a sitting-drop vapor-diffusion method mixing 1 μL of HasApf5 with an equal volume of reservoir solution. For the X-ray diffraction study, crystals were flash-cooled in liquid nitrogen after soaking in a solution containing 70–80% suitable reservoir solution and 20–30% glycerol. The final crystallization condition was as follows:

A 1.0 μL aliquot of a Fe-TPP HasApf5 solution [30 mg mL^−1^ in 0.1 M CHES–KOH buffer (pH 9.5)] was mixed with equal volumes of the reservoir solution [0.1 M Tris–HCl (pH 7.0), 1.0 M potassium sodium tartrate, and 0.2 M Li_2_SO_4_].

### Data collection and structure refinement

X-ray diffraction data was recorded on the beamline BL26B1 equipped with EIGER X 4M (Dectris) at SPring-8 (Hyogo, Japan) with a wavelength of 1.000 Å at 100 K. The Fe-TPP HasApf5 dataset was processed using XDS.^38^ Initial phase was determined by molecular replacement with MOLREP.^39,40^ The SWISS-MODEL structure of HasApf5 with heme was modeled from the crystal structure of holo HasAp (PDB ID: 3ELL^41^) and used as a search model for MOLREP.^40^ Model building and refinement were performed by using Coot^42^ and REFMAC5.^43^ Due to the presence of pseudo-merohedral twinning in the FeTPP-HasApf5 dataset, intensity-based twin refinement was conducted using REFMAC5.^44^ The final refinement converged to a twin fraction of 0.499 for the operator -*l*,-*k*,-*h*. For the refinement process of the FeTPP-HasApf5 structure, the geometry restraints of TPP were generated by PRODRG.^45^ Protein figures were depicted by using PyMOL2. The final refinement statistics are summarized in Table S3.†

### Analysis of multimerization of Fe-TPP-phen HasApf5 with metal ions using SEC

The solution of Fe-TPP-phen HasApf5 concentrated by Amicon Ultra (Merck Millipore, 10 kDa cutoff) was mixed with the metal ions solution (NiCl_2_, CuCl_2,_ or FeCl_2_) in 50 mM CHES–KOH (pH 9.5). The final concentration of Fe-TPP-phen HasApf5 was prepared to be 200 μM. A 100 μL of Fe-TPP-phen HasA solution was loaded onto the SEC (Superdex 200 increase 10/300 GL column; GE Healthcare, Cytiva) equilibrated with 50 mM CHES–KOH (pH 9.5) and then eluted at the flow rate of 0.5 mL min^−1^.

### Model building of Fe-TPP-phen HasApf5 Ni dimer

To simplify the calculation to build the model structure, the metal center of Fe-TPP moiety was changed to Ga^3+^. After creating a rough model structure of Fe-TPP-phen using Avogadro,^46^ the charges of the metal center and the porphyrin were prepared in Maestro. Optimization in GFN2-xTB^47^ (tight, water) provided the model structure of Fe-TPP-phen. The Fe-TPP moiety of Fe-TPP-phen was superimposed on Fe-TPP of the crystal structure of Fe-TPP HasApf5 (PDB ID: 8KI0) in PyMOL2. Following the removal of Fe-TPP of the Fe-TPP HasApf5 structure, the model structure of HasApf5 with Fe-TPP-phen was built. The hydrogen atoms of the HasApf5 moiety were built using PROPKA in Maestro. The His83 was set in the HIP conformation, and the charge of the oxygen atom on the side chain of Tyr75 was set as −1. Optimization in GFN-FF^48^ (loose, water) provided the model structure of HasApf5 with Fe-TPP-phen. Two of the resulting monomeric structures were superimposed on the crystal structure of Ni(phen)_2_(H_2_O)_2_ (CSD ID: RAGSEA or UMOWOJ) along the phen moieties. In this process, we modeled six different conformations based on the isomers of the Ni-phen complex (*Δ*–*Λ* isomers of Ni(phen)_2_(H_2_O)_2_ complex and *cis*–*trans* isomers based on the positional relationship of the amide groups of Fe-TPP-phen). Following the removal of phen of Ni(phen)_2_(H_2_O)_2_ structure, the model structure of Fe-TPP-phen HasApf5 dimer coordinating to a Ni ion was built and finally optimized by GFN-FF^48^ (loose, water) (*Δ-cis*.pdb, *Δ-trans*1.pdb, *Δ-trans*2.pdb, *Λ-cis*.pdb, *Λ-trans*1.pdb, *Λ-trans*2.pdb).

### SEC-SAXS

Small-angle X-ray scattering combined with size-exclusion chromatography, SEC-SAXS was conducted at the beamline BL-10C of the Photon Factory^49^ (Tsukuba, Ibaraki, Japan). The X-ray wavelength and camera distance were set to 1.000 Å and 2080.5 mm, respectively. The X-ray exposure part of the stainless-steel sample cell was covered with 0.02 mm thick quartz glass with an optical path length of 1 mm. 408 SAXS 2D image data were collected using a PILATUS3 2M detector (Dectris) with an exposure time of 20 seconds and an exposure interval of 0.01 seconds. To estimate the sample concentration during X-ray exposure, the same sample cell was used in conjunction with a fiber spectrophotometer, QE65pro (Ocean Insight) to measure UV-vis absorption spectra in the wavelength range of 200 to 800 nm. The incident UV-vis light is guided by the optical fiber at a 45-degree angle to the sample cell. UV-vis absorption spectra were collected with 1 second integrations at 10 seconds intervals for 816 data. For SEC, a high-performance liquid chromatography (HPLC) system, Prominence-i (Shimadzu) equipped with a Superdex 200 increase 10/300 GL column (Cytiva) and equilibrated with a buffer of 50 mM CHES–KOH, 5% Glycerol, pH 9.5 was employed. The sample was dissolved in the same buffer and adjusted to an initial concentration of 8 mg mL^−1^, and 0.44 mL of the solution was injected into the HPLC system. The flow rate during peak elution measurements was set at 0.05 mL min^−1^. All 2D SAXS intensities were azimuthally averaged into one dimension for the scattering vector, *Q* (=4π sin *θ*/*λ*, Å^−1^) where 2*θ* is the scattering angle and *λ* is the measurement X-ray wavelength. Background subtraction was performed using an average of 20 SAXS intensities measured for the buffer region before peak elution. Subsequently, the SAXS intensities were normalized to the absolute scale using water as the standard. These data reduction processes were executed using SAngler.^50^ The acquired SAXS data and UV-vis absorption spectra were processed with MOLASS.^51^ The results of the automated Guinier analysis by MOLASS are presented in ESI Fig. S10a,† where the radius of gyration, *R*_g_ around the peak shows a convex downward distribution, suggesting the presence of interparticle interference effects in the scattering intensity. Therefore, MOLASS generated a SAXS profile with the interparticle interference effect removed using the data between 227th and 286th frames (Fig. S10b†), and a Guinier plot analyzed by AUTORG in ATSAS^52^ is displayed in Fig. S10c.† The presence of a sufficiently long linear region for the Guinier approximation suggests that the interparticle interference effects were effectively eliminated by MOLASS. A dimensionless Kratky plot is shown in Fig. 5e and S10d.† A peak of height 3/*e* typically appears at *QR*_g_ = √3 in the case of a compact spherical structure.^53^ The *P*(*r*) function is calculated using PRIMUSqt in ATSAS,^54^ and a bead model representing the molecular outer shape of the scatterer is generated using the DAMMIF module in PRIMUSqt.^54^ The theoretical SAXS profiles were computed from six atomistic model structures using CRYSOL in ATSAS^55^ and fitted to the experimental profile. The bead model was superimposed on each of the six atomistic model structures using CIFSUP in ATSAS.^54^ Since the *Λ-trans*1 structure had the lowest *χ*^2^ value indicating the agreement with the experimental profile as shown in Table S2,† the remaining five model structures were superimposed on *Λ-trans*1 structure using BIOVIA Discovery Studio before the CIFSUP calculation. Detailed information regarding the SEC-SAXS experiments and analyses are summarized in Table S2,† and these data are deposited in the Small Angle Scattering Biological Data Bank (SASBDB) under the ID of SASDTK5. The graphs in Fig. 5b and e, S10 and S13† were produced using Igor Pro 9 (WaveMetrics). The bead model and six atomistic model structures were drawn using Chimera,^56^ with the resolution of the surface density map derived from the bead model set to 9.8.

## Conflicts of interest

There are no conflicts to declare.

## Supplementary Material

RA-014-D4RA01042F-s001
