# Supplementary material for: Heme-substituted protein assembly bridged by synthetic porphyrin: achieving controlled configuration while maintaining rotational freedom
Source: RSC Adv. 2024 Mar 15;14(13):8829–36. doi: 10.1039/d4ra01042f (PMC10941265; doi:10.1039/d4ra01042f)
Supplement: RA-014-D4RA01042F-s001 [file RA-014-D4RA01042F-s001.pdf]

Electronic Supplementary Material (ESI) for RSC Advances.
This journal is © The Royal Society of Chemistry 2024

Material

All chemical reagents were purchased from commercial sources (e.g. Wako, TCI, and Aldrich) and used without further purification until otherwise noticed.

Instruments

UV-Vis spectra were recorded on a UV-2600 PC spectrophotometer (Shimadzu). ^{1}H NMR (400 MHz) spectra were recorded on ECS400 Delta spectrometer (JEOL), and chemical shifts were reported as the delta scale in ppm relative to $CHCl_3$ (δ 7.26 ppm) for ^{1}H NMR. MALDI-TOF MS spectra were collected using ultrafleXtreme (Bruker). ESI-MS spectra were recorded on micrOTOF II (Bruker) using the positive mode ESI-TOF method.

Construction of an expression system of HasA

The expression system of HasAp was constructed according to published procedures.[1, 2] To construct the expression system of HasApf5, the plasmid pTAKN-2 coding synthetic *hasApf5* gene (pTAKN-2-*hasApf5*) was purchased from Eurofins Genomics. The codon of *hasApf5* gene was optimized for expression in *E. coli* strain K-12. The gene sequence was as follows.

ATGTCGATCTCGATCTCGTATAGCGCGACTTATGGCGGCAACACGGTTGCTCAGTATCTTACCGACTGGTCTGCGTA
CTTTGGCGATGTGAACCATCGCCCAGGAGAAGTGGTGGATGGCACCAACACTGGTGGCTTCAACCCTGGCCCGTTTG
ACGGTACCCAGTATGCCATTAAGTCCACAGCGTCTGATGCTGCCTTTGTAGCCGATGGGAATCTGCACTACACCCTG
TTCAGCAATCCCTCTCATACGTTGTGGGGTAGCGTGGATACGATCTCACTTGGCGATACACTGGCTGGAGGGTCAGG
TAGCAACTACAACCTCGTTTCACAGGAAGTCTCCTTTACCAATCTGGGGCTGAACTCCCTGAAAGAGGAAGGCCGTG
CGGGCGAAGTGCACAAAGTGGTCTATGGCCTGATGAGTGGTGACAGTAGTGCCTTGGCCGGTGAGATTGACGCGTTA
CTGAAAGCGATTGACCCGAGCCTCAGCGTGAATTCGACCTTCGACGATCTGGCAGCGGCAGGAGTCGCGCATGTAAA
TCCGGCAGCG**<u>GCAGCTGCAGCCGATGTAGGTCTGGTTGGTGTTCAAGATGTCGCACAAGATTGGGCCTTAGCCGCG</u>**T
AA

For crystal growth of HasApf5, the C-terminal fragment of HasApf5 (Ala184–Ala205 residues), which is predicted as the secretion signal peptide, was truncated. The signal peptide of HasApf5 was estimated based on the previously reported signal peptide of HasA from *Pseudomonas aeruginosa* PAO1 (HasAp)[3]. The truncated *hasApf5* gene fragment, *hasApf5*(T), was amplified using KOD–Plus–Neo (Toyobo), pTAKN-2-*hasApf5* as the template DNA, and following two primers, 5'- CCA<u>GGATCC</u>**ATG**TCGATCTCGATCTCGTATAG-3' (*Bam*HI site underlined, *hasApf5*(T) start codon in bold) and 5'- CTC<u>AAGCTT</u>**TTA**CGCTGCCGGATTTACATGC-3' (*Hin*dIII site underlined, complement of *hasApf5*(T) stop codon in bold). The truncated bases of *hasApf5* gene were shown as **<u>bold</u>** in the above gene sequence. The amplified fragment was ligated into the plasmid pQE30t[4]. The constructed plasmid, pQE30t-*hasApf5*(T), was then introduced into *E. coli* strain M15.

Expression and purification of apo HasA (HasAp or HasApf5)

Purified heme-free HasA (apo HasA) was prepared according to published procedures.[1, 2] *E. coli* cells expressing HasA were suspended in an H buffer (20 mM sodium phosphate, 20 mM imidazole, 500 mM NaCl, pH 7.4) and disrupted by ultrasonicator at 4°C. After removing cell debris by centrifugation at 17,500 rpm for 30 min (KUBOTA AG-508R angle rotor), the supernatant containing HasA was loaded on Ni-affinity column chromatography (cOmplete His-Tag Purification Resin; Roche). The weakly adsorbing proteins to the Ni-affinity column were washed out using H buffer, and specific binding proteins including HasA were eluted with H buffer containing 200 mM imidazole. The resulting protein solution was treated with thrombin to remove the His-tag on HasA and dialyzed against a phosphate-buffered saline (PBS; 140 mM NaCl, 2.7 mM KCl, 10 mM Na_2HPO_4, 1.8 mM KH_2PO_4, pH 7.3) solution overnight. This sample was further purified by the same Ni-affinity column chromatography equilibrated with PBS solution. The purified HasA solution was denatured by acetone including 0.2 % (w/v) conc. HCl to remove heme. The precipitate of HasA was collected by centrifugation and dissolved in a solution of 7 M urea including 100 mM Tris-HCl (pH7.5). The resulting solution was dialyzed against a PBS solution. After overnight dialysis, the solution was concentrated using Amicon Ultra (Merck Millipore, 3 kDa cutoff) and purified by a gel filtration column (HiPrep 16/60 Sephacryl S-200 HR; GE Healthcare, Cytiva) equilibrated with a PBS solution to remove unfolded HasA. The concentration of apo HasA was determined by absorption at 280 nm (HasAp: ε_{280} = 28.6 mM^{-1} cm^{-1},[5] HasApf5: ε_{280} = 28.5 mM^{-1} cm^{-1} estimated via a Pierce™ BCA Protein Assay Kit (Thermo Scientific) using HasAp as the protein standard). The purified apo HasA solution was frozen by liquid nitrogen and stored at –80 °C until use.

Preparation of HasApf5 with Fe-salophen

The salophen ligand and its iron complex (Fe-salophen Cl) were prepared according to a method described in the literature.[1, 6] A methanol solution of Fe-salophen·Cl was added to apo-HasApf5 in a PBS solution on ice and gently stirred at 4 °C. Following overnight dialysis of a mixture of Fe-salophen/HasApf5 in PBS, excess Fe-salophen was removed by filtration through a PVDF membrane (0.22 µm, Millipore). The resulting HasApf5 solution was loaded on an anion exchange column (HiTrap capto DEAE; GE Healthcare, Cytiva) equilibrated with buffer A [50 mM Tris-HCl (pH 7.5)] and washed by 1 column volume of the buffer A containing 10%(v/v) of buffer B [50 mM Tris-HCl, 800 mM NaCl (pH 7.5)]. The binding proteins to the anion exchange column were then eluted with 16 column volumes of a linear gradient from 10% to 80% as the ratio of buffer B, and we collected the pure Fe-salophen coordinating HasApf5 solution. After buffer exchange to PBS solution using desalting column (PD-10; GE Healthcare, Cytiva), the HasApf5 with Fe-salophen solution was frozen by liquid nitrogen and stored at 4 °C or –80 °C until use.

Preparation of HasApf5 with Fe-Pc

Fe(II) phthalocyanine (Fe-Pc) was purchased from TCI and used without further purification. Fe-Pc was solubilized in DMSO and then mixed with apo HasApf5 in PBS. Following overnight dialysis of a mixture of Fe-Pc/HasApf5 in PBS, excess Fe-Pc was removed by filtration through a PVDF membrane (0.22 µm, Millipore). The resulting HasApf5 solution was loaded on an anion exchange column (HiTrap capto DEAE; GE Healthcare, Cytiva) equilibrated with buffer A [50 mM Tris-HCl (pH 7.5)] and washed by 1 column volume of the buffer A containing

10%(v/v) of buffer B [50 mM Tris-HCl, 800 mM NaCl (pH 7.5)]. The binding proteins to the anion exchange column were then eluted with 16 column volumes of a linear gradient from 10% to 80% as the ratio of buffer B, and we collected the pure Fe-Pc coordinating HasApf5 solution. After buffer exchange to PBS solution using desalting column (PD-10; GE Healthcare, Cytiva), the HasApf5 with Fe-Pc solution was frozen by liquid nitrogen and stored at 4 °C or –80 °C until use.

Preparation of heme-bound HasApf5 (holo HasApf5).

The purified apo HasApf5 was mixed with a hemin solution (Hemin, from Porcine; Wako) to form a 1 : 1 complex. The HasApf5/heme mixture was purified via size exclusion chromatography (HiPrep 16/60 Sephacryl S-200 HR; GE Healthcare) equilibrated with PBS buffer. The Holo-HasApf5 concentration was quantified by pyridine hemochrome assay[7]. The extinction coefficient of Holo-HasApf5 was calculated to be 132.8 mM^{-1} cm^{-1} from the characteristic absorption at 407.5 nm.

Analysis of Fe-TPP HasApf5 stability via UV-Vis spectroscopy

After buffer exchange to 0.1 M KPi (pH 7.0) or 0.1 M CHES-KOH (pH 9.5) using desalting column (PD-10; GE Healthcare, Cytiva), the concentration of Fe-TPP HasApf5 solutions was adjusted to 15 μM. The solutions of Fe-TPP HasApf5 were incubated at 4 °C and UV-Vis spectra were measured every 24 h after centrifugation (11,000 rpm, 10 min).

Circular Dichroism (CD) Spectroscopy

The thermal stability of HasApf5 capturing Fe-TPP was analyzed by CD spectroscopy (J-1500; JASCO). Molar ellipticity at 222 nm was measured as a function of temperature from 35 °C to 95 °C at increments of 1.0 °C min^{-1} controlled by a Peltier thermostat. Temperatures upon thermal denaturation (T_m) were determined by a previously reported method.[8]

Crystallization of HasApf5 capturing heme

Purified holo HasApf5 was exchanged into a crystallization buffer [0.1 M KPi buffer (pH 7.0)] via a PD-10 desalting column (GE Healthcare, Cytiva). The eluate was concentrated via an Amicon Ultra filter 3 kDa cutoff (Merck Millipore). The final concentration was determined by UV-Vis spectroscopy. The crystallization condition was obtained from screening tests using Wizard Classic 1 and 2 (Rigaku). Crystals were grown at 20 °C using a sitting-drop vapor-diffusion method mixing 1 μL of HasApf5 with an equal volume of reservoir solution. For the X-ray diffraction study, crystals were flash-cooled in liquid nitrogen after soaking in a solution containing 70–80% suitable reservoir solution and 20–30% glycerol. The final crystallization condition was as follows:

Crystallization condition of holo HasApf5.

A 1.0 μL aliquot of a holo HasApf5 solution [50 mg mL^{-1} in 0.1 M KPi buffer (pH 7.0)] was mixed with equal volumes of reservoir solution [0.1 M sodium acetate buffer (pH 4.5), 2.5 M NaCl, and 0.2 M Li_2SO_4].

Data collection and structure refinement of holo HasApf5

X-ray diffraction data was recorded on the beamline BL26B1 equipped with EIGER X 4M (Dectris) at SPring-8 (Hyogo, Japan) with a wavelength of 1.000 Å at 100 K. Reflection data of holo-HasApf5 was integrated by the program XDS[9] and scaled with Aimless[10-12]. Initial phases were determined by molecular replacement with MolRep[13, 14]. The SWISS-MODEL structure of HasApf5 with heme was modeled from the crystal structure of holo HasAp (PDB ID: 3ELL[15]) and used as a search model for MolRep[14]. Model building and refinement were performed by using Coot[16] and REFMAC5[17]. All protein figures were depicted by using PyMOL2. The final refinement statistics are summarized in Table. S3.

Energy diagram of the Fe-TPP-phen HasApf5 Ni dimer.

The optimized model structure of Fe-TPP-phen HasApf5 Ni dimer (*Λ*-trans1) was used. Changing a dihedral angle between the phenyl group and the amide bond of Fe-TPP-phen by 5 degrees using PyMOL2, we created model structures that simulate the rotation of the dihedral angle. The total energies of these model structures were calculated with GFN-FF[18] (single-point energy calculation, water) to create an energy diagram showing the rotation of the dihedral angle.

Synthesis of TPP derivatives

[5-(4-carboxyphenyl)-10,15,20-triphenyl]porphyrin (TPP-COOH) was synthesized according to the published procedures[19] and characterized by ^{1}H NMR and MALDI-TOF MS. ^{1}H NMR ($CDCl_3$, 400 MHz) δ = 8.86-8.84 (m, 8H), 8.44 (d, J = 7.3 Hz, 2H), 8.24-8.20(m, 8H), 7.79-7.73 (m, 9H), -2.78 (s, 2H), MALDI-TOF MS: m/z calced. for C45H30N4O2 $[M+H]^+$ 659.2, found 659.3.

<u>N-(1,10-phenanthrolin-5-yl)-4-(10,15,20-triphenylporphyrin-5-yl)benzamide (TPP-phen)</u>

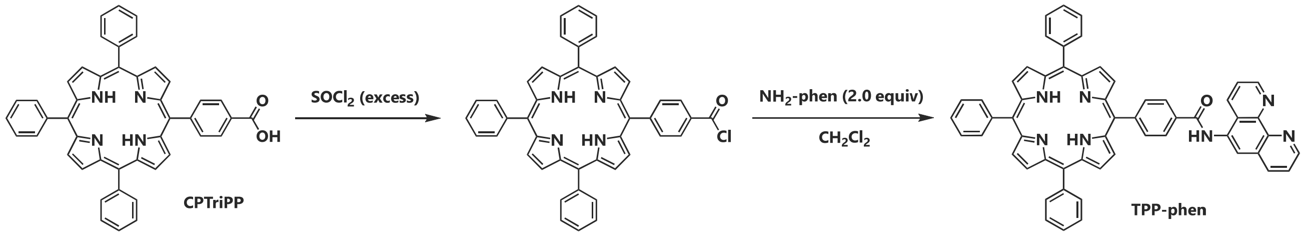

A solution of CPTriPP (23.8 mg, 36.1 μmol) in thionyl chloride (1.20 ml) was refluxed in an argon atmosphere for 2 h following the removal of excess reagent under reduced pressure. The resulting green solid was redissolved in CH_2Cl_2 (7.2 ml), and 5-amino-1,10-phenanthroline (NH_2-phen) (14.3 mg, 72.3 μmol) was added. The mixture was refluxed in an argon atmosphere overnight. After adding CH_2Cl_2 with 3% triethylamine (10 ml), the organic phase was washed with saturated aqueous $NaHCO_3$ solution and water, dried over Na_2SO_4, and removed the solvent under reduced pressure. The residue was purified by alumina column chromatography (CH_3Cl : Hexane = 3:1 ~ 1:0) to give the pure product. TPP-phen was given as a purple solid (20.3 mg, 21.9 μmol, 61% yield). ^{1}H NMR ($CDCl_3$, 400 MHz) δ = 9.28 (d, J = 2.4, 1H), 9.21 (d, J = 3.9 Hz, 1H), 8.91-8.74 (m, 8H), 8.61-8.22(m, 14H), 7.78-7.67 (m, 11H), -2.77 (s, 2H), MALDI-TOF MS: m/z calced. for C57H37N7O $[M+H]^+$ 836.3, found 836.4.

Fe(III)-Cl-N-(1,10-phenanthrolin-5-yl)-4-(10,15,20-triphenylporphyrin-5-yl)benzamide (Fe-TPP-phen)

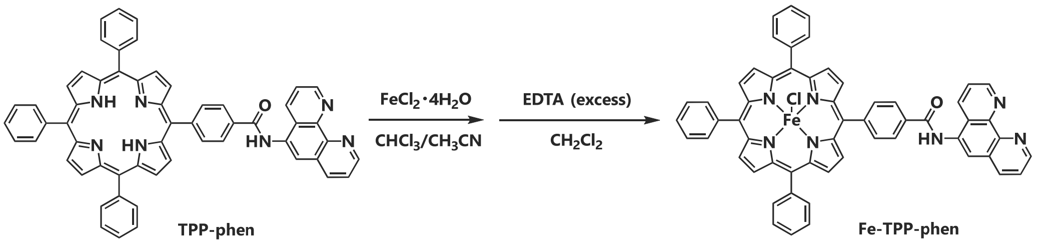

TPP-phen (17.1 mg, 20.5 μmol) and iron(II) chloride tetrahydrate (32.7 mg, 164 μmol) were dissolved in $CHCl_3$ (2.96 ml) and CH_3CN (5.93 ml). After the bubbling of argon, the solution was refluxed for 1.5 h. CH_2Cl_2 (10 ml) was added, and the organic phase was washed with 2 M aqueous HCl solution and water. The resulting solution was dried over Na_2SO_4 and removed the solvent under reduced pressure. The residue was redissolved with CH_2Cl_2 (4.5 ml) and added EDTA buffer (4.5 ml) prepared by mixing $Na_2(H_2EDTA)_2H_2O$ (372 mg), sodium acetate (0.22 g) and acetic acid (0.21 mL) in water (100 mL).[20] The solution was stirred for 24 hours at room temperature. The organic phase was separated, washed with 4% sodium bicarbonate solution, 2 M aqueous HCl solution, and water, dried over Na_2SO_4, and removed the solvent under reduced pressure. Fe-TPP-phen was given as a dark brown solid (14.5 mg, 15.7 μmol, 77% yield). MALDI-TOF MS: m/z calced. for C57H35ClFeN7O $[M{-}Cl]^+$ 889.2, found 889.8.

Supplementary Figures

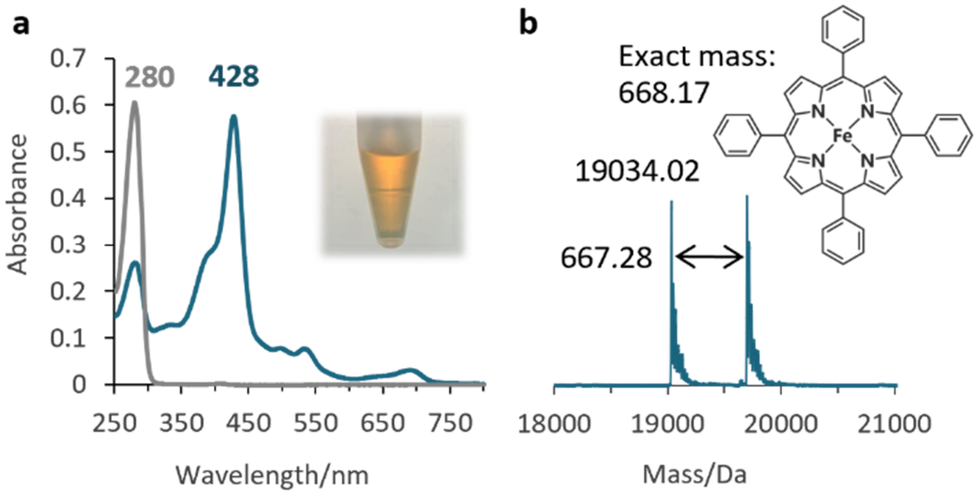

Fig. S1 (a) UV-Vis spectra of Fe-TPP HasApf5 in 50 mM CHES-KOH pH 9.5 (blue) and apo HasApf5 in PBS buffer (gray). The picture of the solution of Fe-TPP HasApf5 in a plastic container is shown as an inset. (b) Positive-mode ESI-TOF mass spectrum of Fe-TPP HasApf5 in 5 mM ammonium acetate buffer. Calculated molecular weight of complex-free HasA (apo HasApf5): 19034.49.

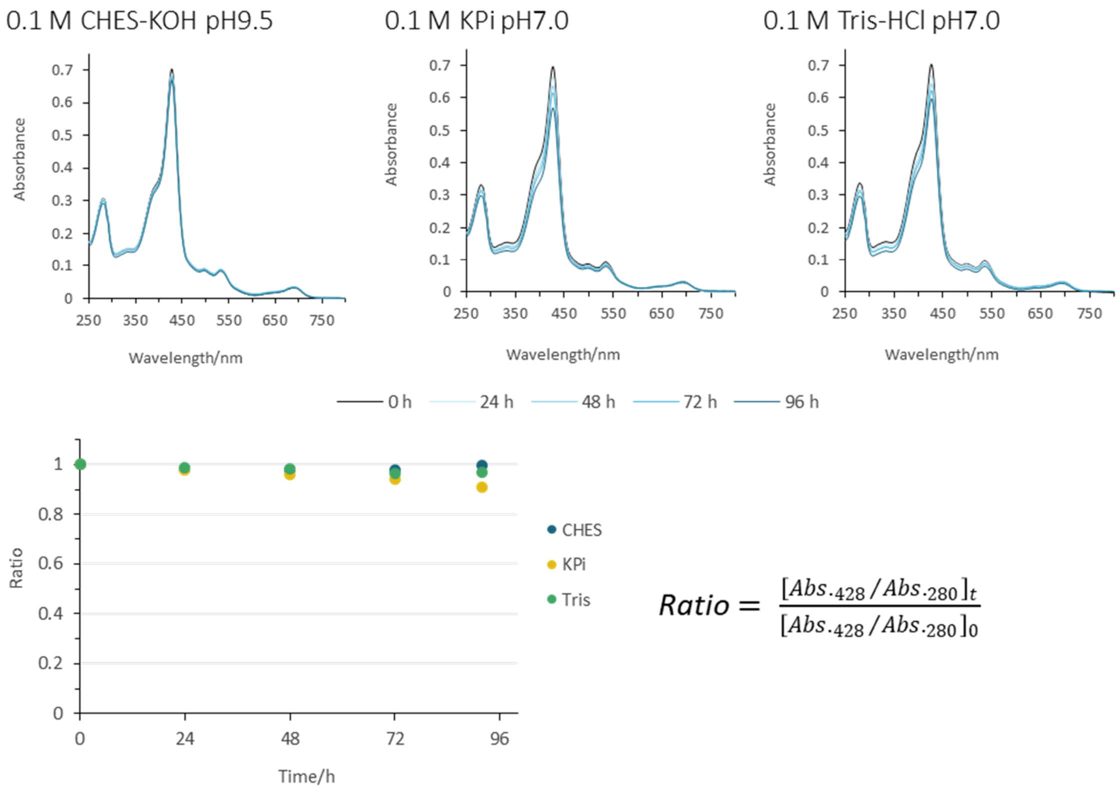

Fig. S2 Time-dependent changes in UV-Vis spectra of Fe-TPP HasApf5 in alkaline and neutral buffer. The graph shows the changes in the ratio of Fe-TPP bound form to the remaining HasApf5.

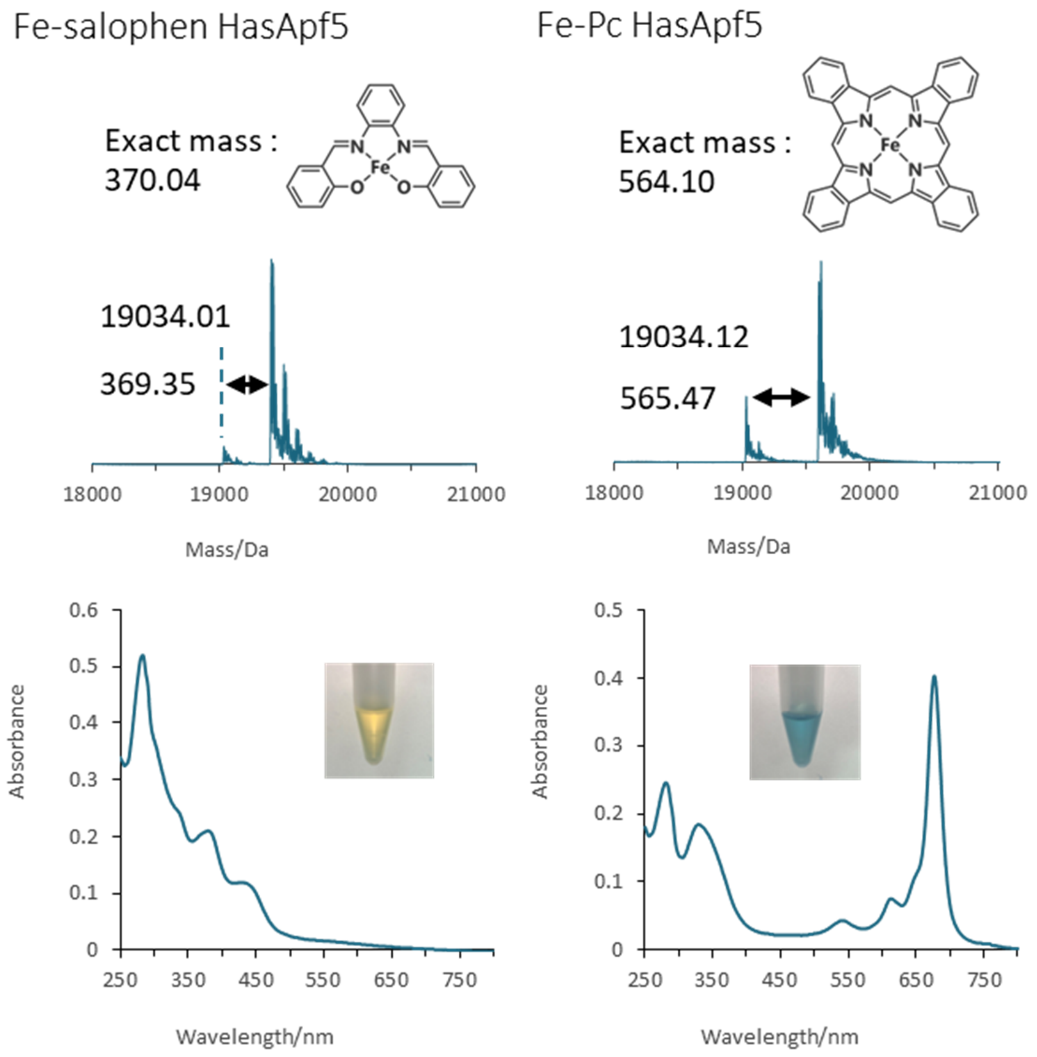

Fig. S3 Positive-mode ESI-TOF mass spectra and UV-Vis spectra of HasApf5s with Fe-salophen, or Fe-Pc. The structures of the metal complexes and pictures of the protein solutions in plastic containers are shown as insets.

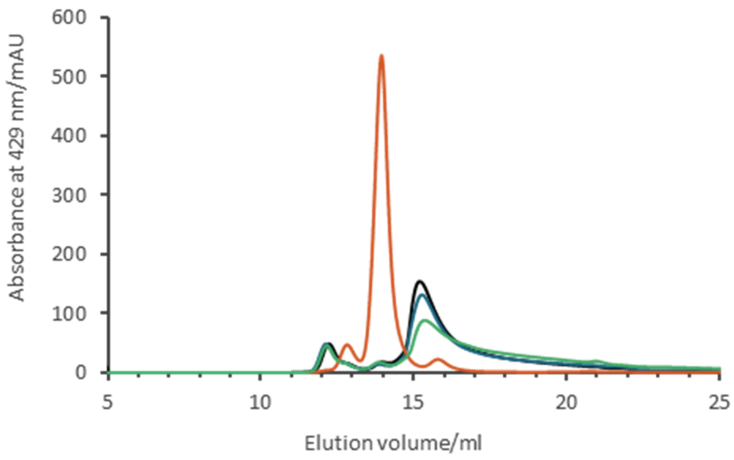

Fig. S4 SEC analysis of Fe-TPP-phen HasApf5 (black), with 0.5 equiv. of Ni^{2+} (red), Fe^{2+} (blue), and Cu^{2+} (green).

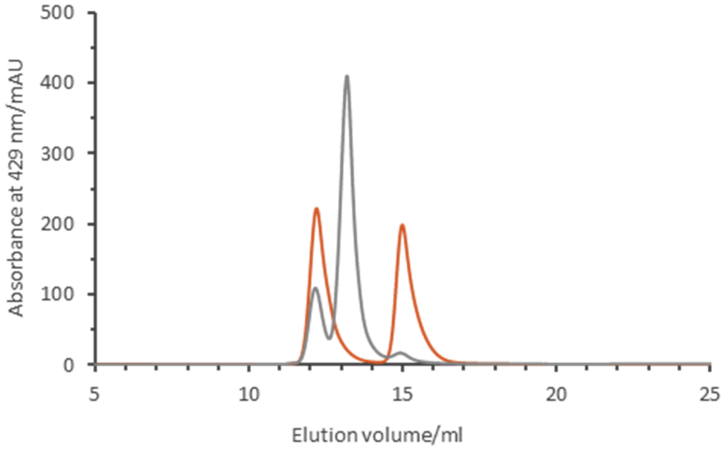

Fig. S5 SEC analysis of the Fe-TPP-phen HasApf5 with Ni^{2+} and EDTA. Following incubation of the solution of Fe-TPP-phen HasApf5 with Ni^{2+} (0.5 equiv.) for 24 hours at 4 °C, then 50 equiv. EDTA was added, and the mixture was analyzed after incubation for 24 hours. Fe-TPP-phen HasApf5 with Ni^{2+} and EDTA (red), and only Ni^{2+} (gray).

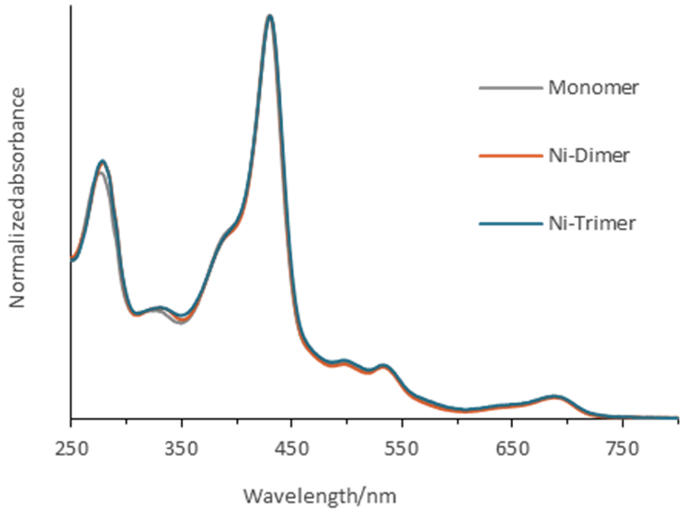

Fig. S6 UV-Vis spectra of Fe-TPP-phen HasApf5 monomer (gray), dimer (red), and trimer (blue). These spectra were obtained by measuring each fraction of SEC.

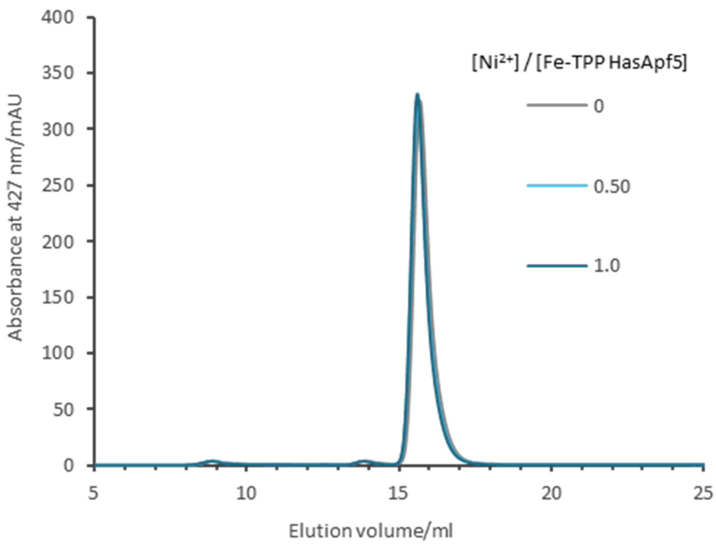

Fig. S7 SEC analysis of Fe-TPP HasApf5 after Ni^{2+} treatment.

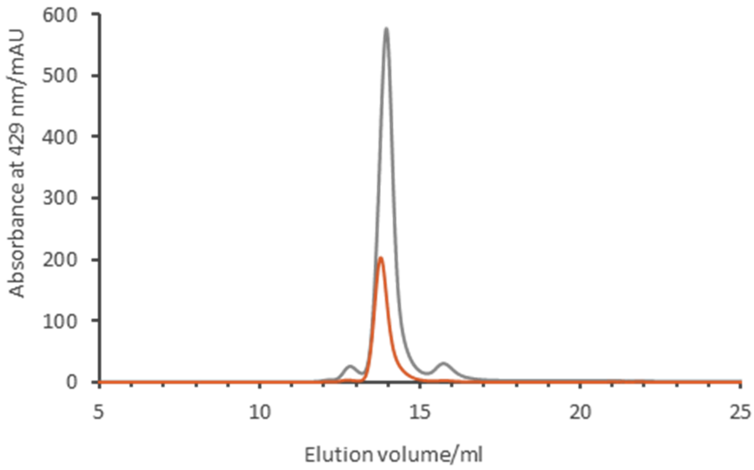

Fig. S8 SEC analysis of the solution of Fe-TPP-phen HasApf5 with Ni^{2+} in 50 mM CHES-KOH pH 9.5. Following incubation of the solution of Fe-TPP-phen HasApf5 with Ni^{2+} (0.5 equiv.) in 50 mM CHES-KOH pH 9.5 for 24 hours, the mixture was analyzed (gray). In addition, the fraction containing the dimer in 50 mM CHES-KOH pH 9.5 was collected and concentrated. Following incubation of the concentrated solution for 24 hours at 4 °C, the solution was analyzed again (red).

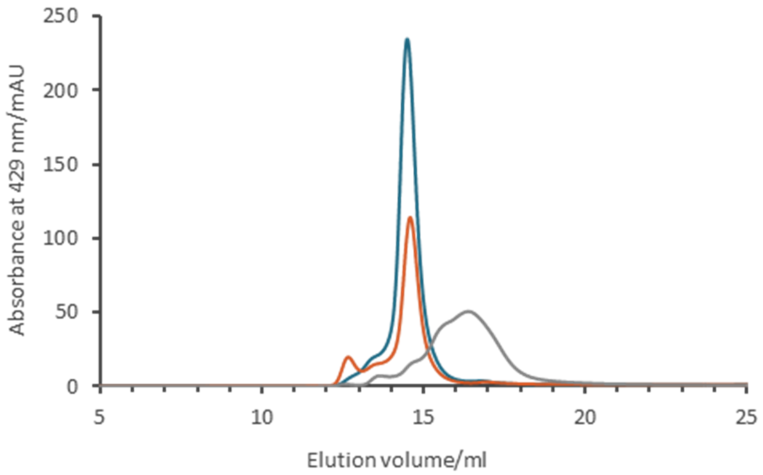

Fig. S9 SEC analysis of the solution of Fe-TPP-phen HasApf5 with Ni^{2+} in 50 mM KPi pH 7.0. Following incubation of the solution of Fe-TPP-phen HasApf5 with Ni^{2+} (0.5 equiv.) in 50 mM CHES-KOH pH 9.5 for 24 hours, the mixture was analyzed (blue). In addition, the fraction containing the dimer in 50 mM KPi pH 7.0 was collected and concentrated. Following incubation of the concentrated solution for 24 hours at 4 °C, the solution was analyzed again (red). Furthermore, the solution of Fe-TPP-phen HasApf5 was also analyzed (gray).

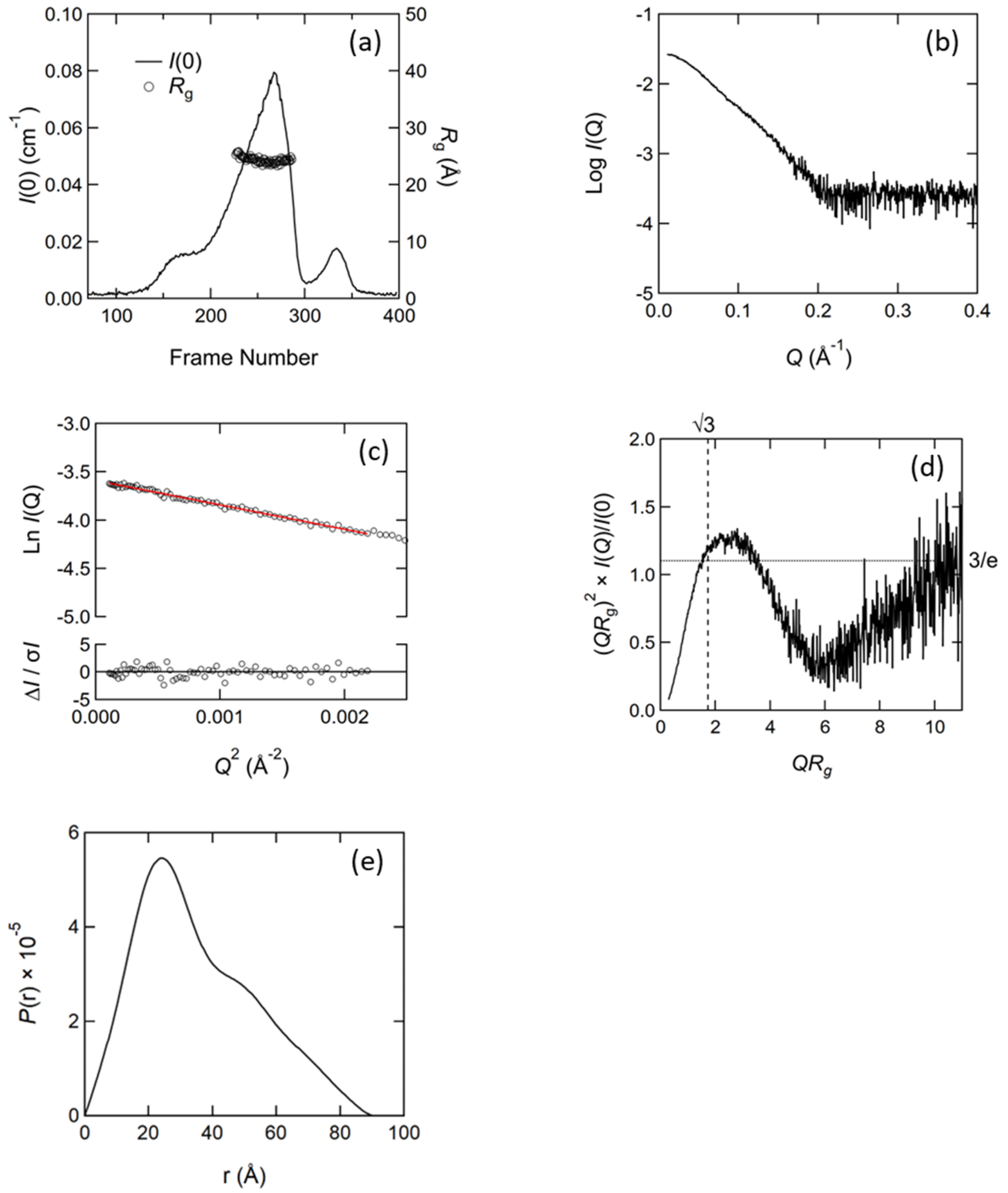

Fig. S10 (a) Distribution plots of $I(0)$ and R_g obtained from Guinier analysis of all SEC-SAXS data. R_g values between 227th and 286th data used to derive the SAXS profile are plotted. (b) Acquired SAXS experimental profile of the resulting dimer. (c) Guinier plot for (b). The red line represents the result of the linear approximation in the Guinier analysis. The residuals of the linear approximation are shown at the bottom of the graph. (d) Dimensionless Kratky plot of (b). (e) P(r) function.

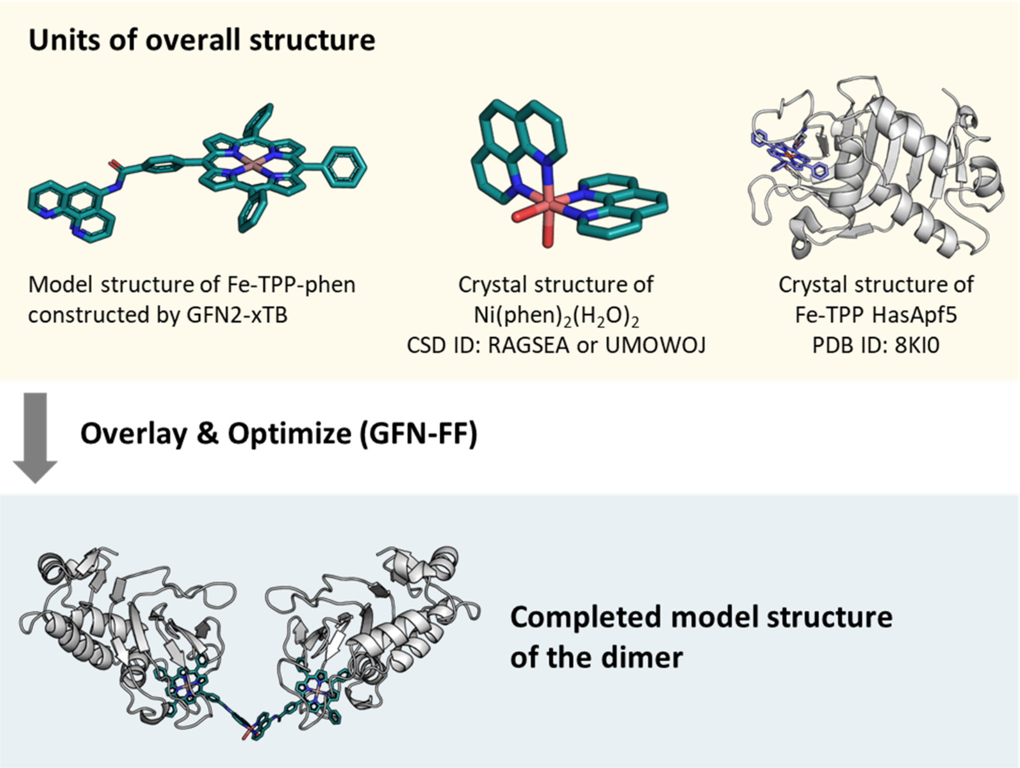

Fig. S11 Scheme of structure prediction of HasApf5 dimers.

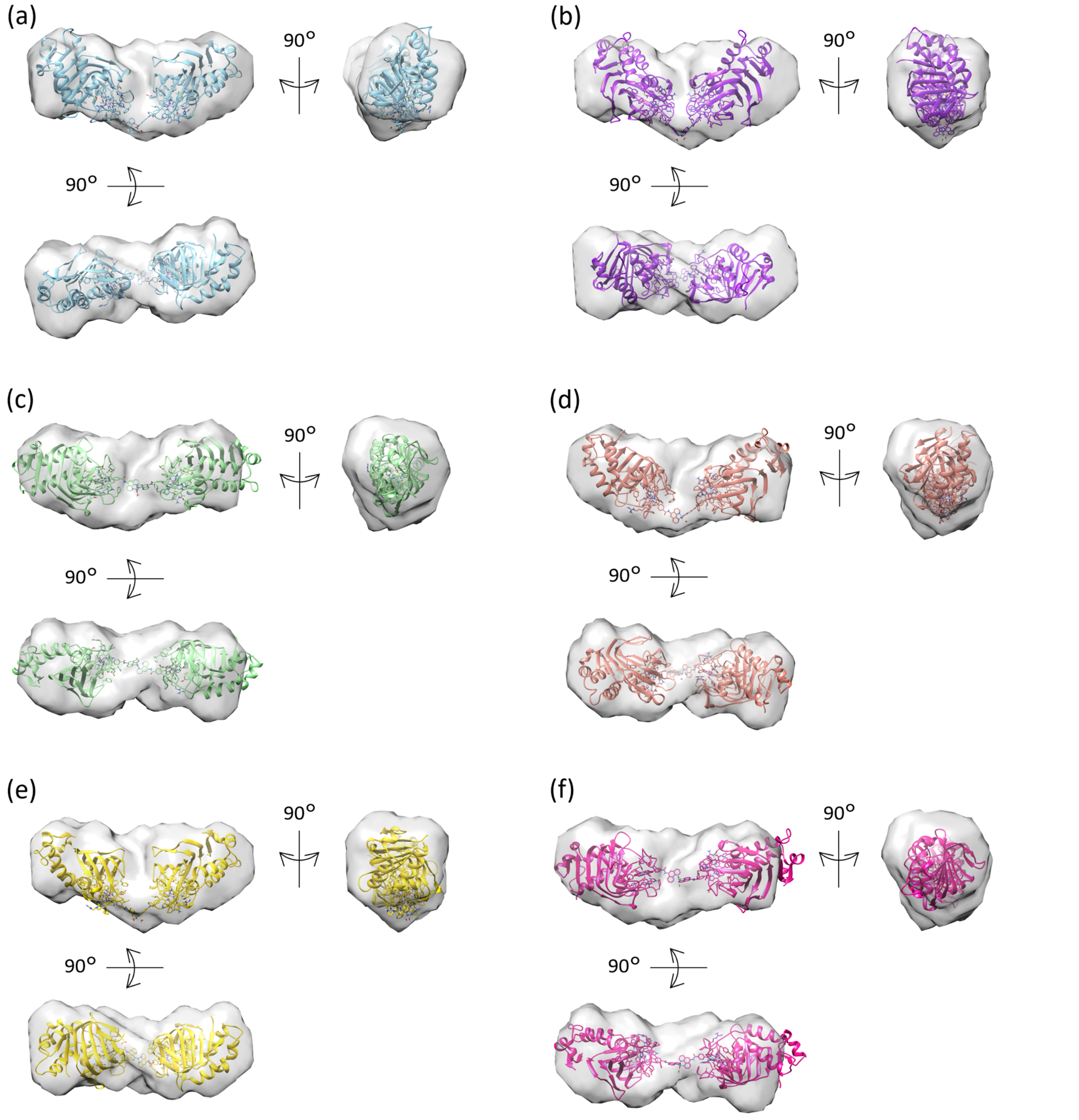

Fig. S12 The bead model was superimposed on each of the sic atomistic models. (a) Δ-cis; (b) Δ-trans1; (c) Δ-trans2; (d) Λ-cis; (e) Λ-trans1; (f) Λ-trans2.

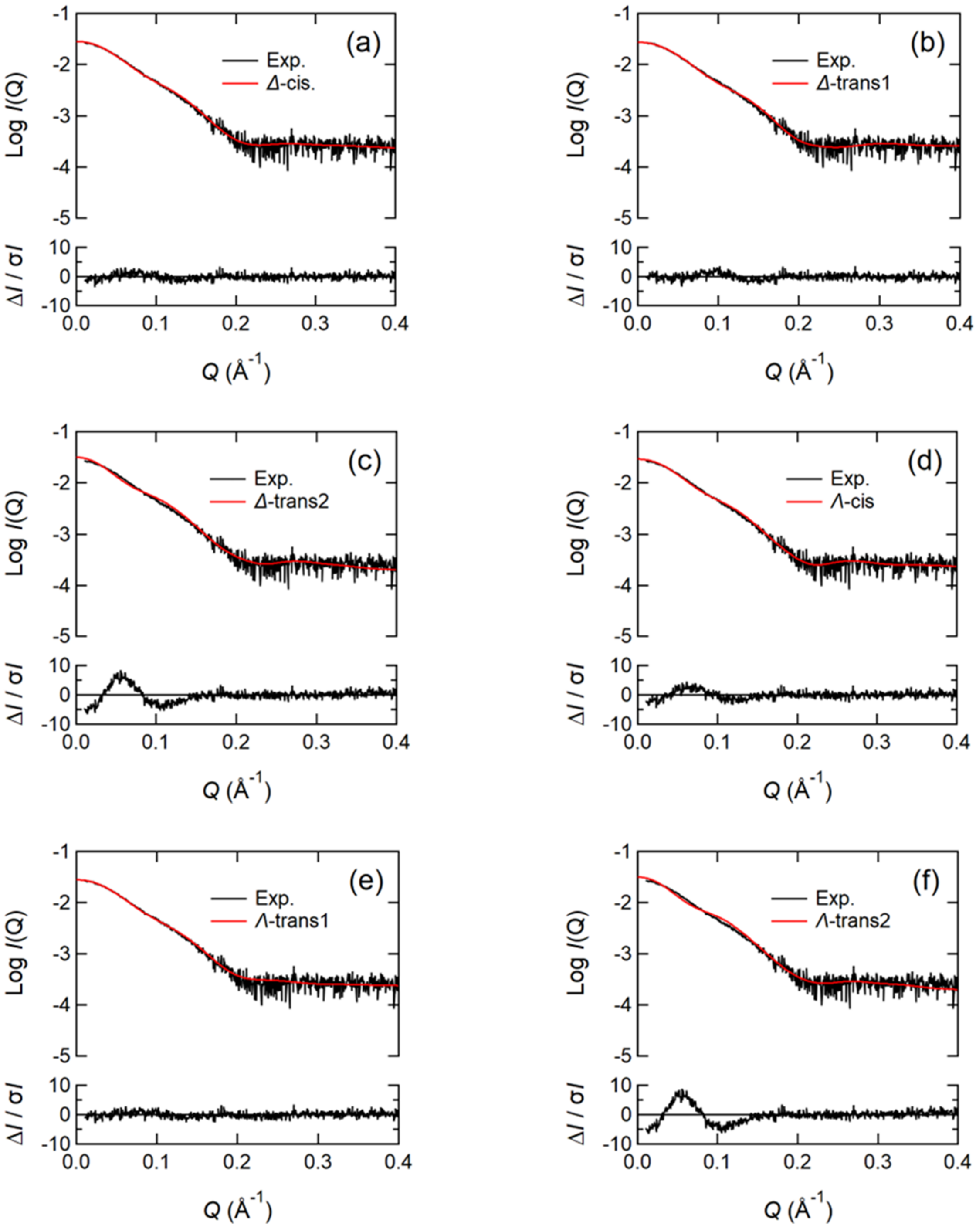

Fig. S13 Theoretical SAXS profiles calculated by CRYSOL (red) fitted to the experimental SAXS profile (black). The residuals between the two profiles are plotted at the bottom of the graph. (a) *Δ*-cis; (b) *Δ*-trans1; (c) *Δ*-trans2; (d) *Λ*-cis; (e) *Λ*-trans1; (f) *Λ*-trans2.

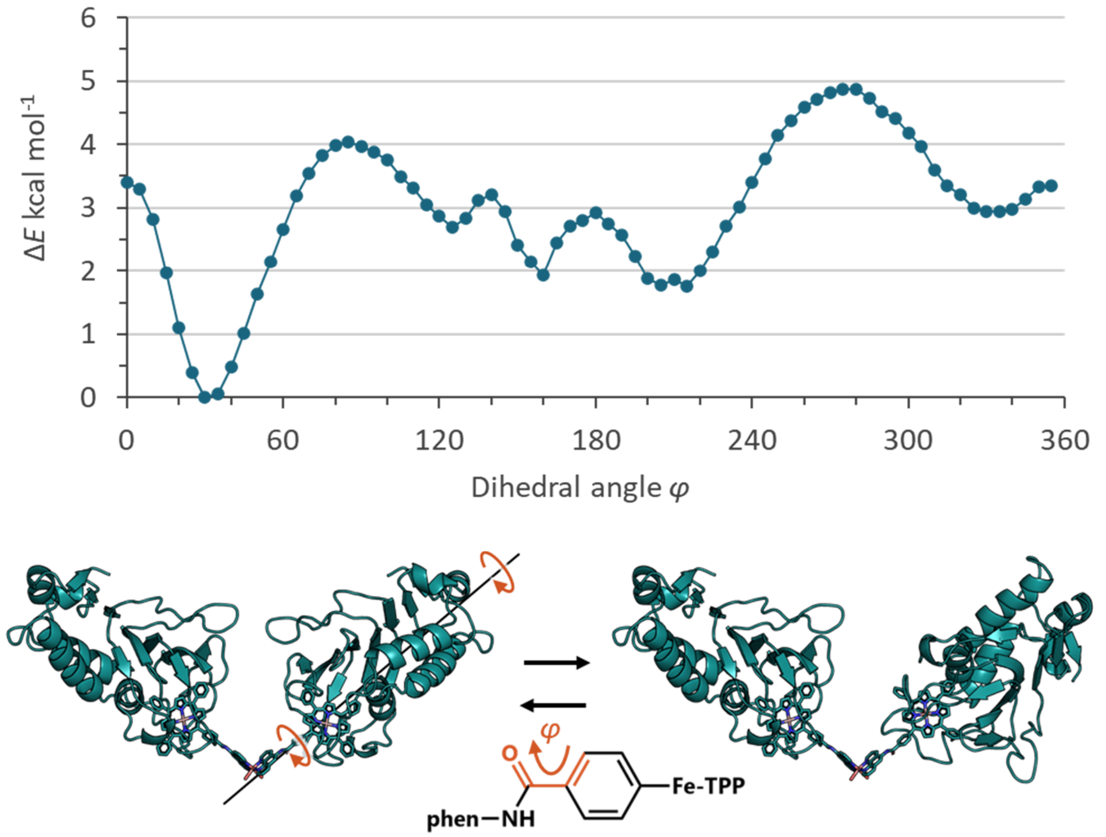

Fig. S14 Energy diagram of Λ-trans1 with changing the dihedral angle between the amide bond and the phenyl group of Fe-TPP-phen.

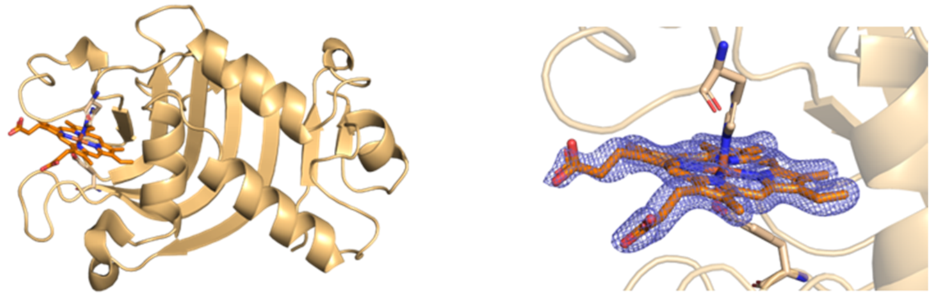

Fig. S15 Crystal structure of holo HasApf5 and enlarged view of the heme-binding site of HasApf5. Polder map omitting heme contoured at 5.0σ (blue mesh).

```
HasAp      1    MSISISYSTTYSGWTVADYLADWSAYFGDVNHRPGQVVDGSNTGGFNPGPFDGSQYALKS  60
                MSISISYS TY G TVA YL DWSAYFGDVNHRPG+VVDG+NTGGFNPGPFDG+QYA+KS
HasApf-5   1    MSISISYSATYGGNTVAQYLTDWSAYFGDVNHRPGEVVDGTNTGGFNPGPFDGTQYAIKS  60

HasAp      61   TASDAAFIAGGDLHYTLFSNPSHTLWGKLDSIALGDTLTGGASSGGYALDSQEVSFSNLG  120
                TASDAAF+A G+LHYTLFSNPSHTLWG +D+I+LGDTL GG+ S  Y L SQEVSF+NLG
HasApf-5   61   TASDAAFVADGNLHYTLFSNPSHTLWGSVDTISLGDTLAGGSGS-NYNLVSQEVSFTNLG  119

HasAp      121  LDSPIAQGRDGTVHKVVYGLMSGDSSALQGQIDALLKAVDPSLSINSTFDQLAAAGVAHA  180
                L+S   +GR G VHKVVYGLMSGDSSAL G+IDALLKA+DPSLS+NSTFD LAAAGVAH
HasApf-5   120  LNSLKEEGRAGEVHKVVYGLMSGDSSALAGEIDALLKAIDPSLSVNSTFDDLAAAGVAHV  179

HasAp      181  TP-AAAAAEVGVVGVQELPHDLALAA  205
                 P AAAAA+VG+VGVQ++  D ALAA
HasApf-5   180  NPAAAAAADVGLVGVQDVAQDWALAA  205
```

Fig. S16 Comparison of amino acid sequences between HasAp and HasApf5

Supplementary Tables

Table. S1 Comparison of thermostability of HasAs capturing Fe-TPP.

	**Fe-TPP HasAp**[2]	**Fe-TPP HasApf5**
$\boldsymbol{T_m}$**/°C**	62.4	60.5

Table S2. SAS data acquisition, sample details, data analysis, modeling fitting, and software used.

**A. Sample details**	**Fe-TPP-phen HasApf5 with Ni ions**
Organism	*Pseudomonas protegens* Pf-5
Source	*Escherichia coli* M15 expressed
Uniprot ID (residues in construct)	Q4K5N8
Extinction coefficient (A_{280nm}, 0.1%(w/v))	3.448
P.S.V (cm^3 g^{-1})	0.728
Contrast (cm^{-2})	2.842×10^{10}
Theoretical *M* (Da)	39,943
SEC-SAXS column	Superdex 200 increase 10/300 GL (Cytiva)
Initial conc. (mg ml^{-1})	8.0
Injection Volume (μL)	440
Flow rate (ml min^{-1})	0.05
Conc. range (mg ml^{-1})	1.49-3.10
Solvent composition	50 mM CHES-KOH, 5 % Glycerol, pH 9.5
Concentration method	UV-Vis spectroscopy

**B. SAS data collection**	**Fe-TPP-phen HasApf5 with Ni ions**
Instrument	Photon Factory, BL-10C
Wavelength (Å)	1.000
Beam geometry (μm)	V370 × H890, Bent cylindrical mirror + 2 slits + 1 pinhole
Camera length (mm)	2080.5
Q range ($Å^{-1}$)	0.0104-0.3994
Absolute scaling method	Comparison with scattering from 1 mm pure H_2O
Expo. time (sec), No. of frames	20, 408
Sample path length (mm)	1
Sample temperature (K)	293

Normalization	To transmit intensity by beam-stop counter
Method for monitoring radiation damage	Data frame-by-frame comparison

**C. Software employed for SAS data reduction, analysis, and interpretation**	
SAS data processing	*SAngler 2.1.65, MOLASS 1.0.12*
Extinction coefficient estimate	*ProtParam*
Calculation of contrast and PSV values	*MULCh*
Basic analyses: (Guinier, P(r), Porod Volume)	*MOLASS 1.0.12, PRIMUSqt from ATSAS 3.2.1*
Shape modeling	*DAMMIF module of PRIMUSqt from ATSAS 3.2.1*
Superimposition between model structures	*BIOVIA Discovery Studio, CIFSUP from ATSAS 3.2.1*
Atomistic structure modeling	*CRYSOL from ATSAS 3.2.1*
Drawing of the surface density map from the bead model and the atomistic model structures	*Chimera 1.17.3*

**D. Structural parameters**	**Fe-TPP-phen HasApf5 with Ni ions**
Guinier Analysis	
$I(0)$ (cm^{-1})	0.02760 +/- 0.00012
R_g (Å)	27.5 +/- 0.2
Q-range (Å^{-1}), $Q \times R_g$	0.0104-0.0468, 0.29-1.29
$P(r)$ analysis	
$I(0)$ (cm^{-1})	0.02774 +/- 0.00009
R_g (Å)	28.2 +/- 0.2
D_{max} (Å)	91.2
Q-range (Å^{-1})	0.0104-0.2901
Porod volume (Å^{-3}) (ratio V_p/Theoretical M)	54,375 (1.361)
M from $I(0)$ (Ratio to an expected value)	39,391 (0.986)

**E. Shape modeling results**	**Fe-TPP-phen HasApf5 with Ni ions**
DAMMIF (default parameters, Slow mode, 20 calculations)	

Q-range for fitting	0.0104-0.2901					
Symmetry, anisotropy	P1, none					
ICP (standard deviation), clusters	6.14 (4.65), 4					
χ^2 range	0.862-0.863					
Model resolution (Å)	27.4					
DAMMIN (default parameters)						
Q-range for fitting	0.0104-0.2901					
Symmetry, anisotropy	P1, none					
χ^2 value, CORMAP P-value	0.849, 0.996					
Constant adjustment to intensities	1.993×10^{-4}					
Model M (×0.5 V_{model}) (Da)	36,346					
Dummy atom radius (Å)	2.8					
CIFSUP: Superimposition between the bead model and the following structures (Option: Default)						
Atomic model structure	*Δ*-cis	*Δ*-trans1	*Δ*-trans2	*Λ*-cis	*Λ*-trans1	*Λ*-trans2
ICP score	9.50	13.62	15.49	10.93	9.14	16.36

**F. Atomistic structure modeling**	**Fe-TPP-phen HasApf5 with Ni ions**					
Atomistic model structure	*Δ*-cis	*Δ*-trans1	*Δ*-trans2	*Λ*-cis	*Λ*-trans1	*Λ*-trans2
Q-range for all modeling	0.0104-0.3994					
CRYSOL (default parameters, Constant subtraction allowed)						
χ^2 value	1.14	1.17	5.22	1.81	1.03	6.09
Predicted R_g (Å)	29.2	27.5	34.8	30.4	28.7	35.2
Vol (Å)	51,575					
Ra (Å)	1.400					
Dro (eÅ^{-3})	0.043	0.040	0.075	0.050	0.033	0.075

**H. SASBDB ID**	
**Fe-TPP-phen HasApf5 with Ni ions**	SASDTK5

Table. S3 Summary of crystallographic parameters

**Structure title (PDB ID)**	**Fe-TPP HasApf5 (8KI0)**	**Holo HasApf5 (8KI1)**
**Data collection**		
Beamline (SPring-8)	BL26B1	BL26B1
Wavelength (Å)	1.0000	1.0000
Space group	$P12_11$	$P6_5$
Cell dimensions		
a, b, c (Å)	33.56, 158.72, 33.53	99.6, 99.6, 90.47
α, β, γ (Å)	90, 97.58, 90	90, 90, 120
Resolution (Å)	39.68–1.45 (1.48–1.45)	45.24–1.90 (1.94–1.90)
Total no. of reflections	59034 (4042)	835293 (56220)
No. of unique reflections	58633 (5324)	40219 (2570)
R_{merge} (%)	7.6 (41.5)	31.1 (481.8)
Completeness (%)	95.6 (85.3)	100.0 (100.0)
Mean $I/\sigma(I)$	16.1 (3.8)	11.7 (1.7)
$CC_{1/2}$ (%)	99.8 (87.1)	99.8 (58.4)
Multiplicity	6.5 (5.2)	20.8 (21.9)
Wilson B factor (Å^2)	12.4	26.3
**Refinement statistics**		
Resolution (Å)	20.0-1.45	20.0-1.90
No. of monomers per asymmetric unit	2	2
R_{work}/R_{free} (%)	11.9/15.2	15.8/19.3
RMSD bond lengths (Å)	0.0092	0.0087
RMSD bond angles (°)	1.5404	1.2353
No. of atoms		
Proteins	2693	2871
Ligand/Ion	144	120
Water	309	227
Average B-factors (Å^2)		
Protein	13.47	28.89
Ligand/Ion	11.84	36.37
Water	24.48	36.67

RMSD, root-mean-square deviation.

References

1. C. Shirataki, O. Shoji, M. Terada, S.-I. Ozaki, H. Sugimoto, Y. Shiro and Y. Watanabe, *Angew. Chem., Int. Ed.*, 2014, **53**, 2862-2866.
2. Y. Shisaka, E. Sakakibara, K. Suzuki, J. K. Stanfield, H. Onoda, G. Ueda, M. Hatano, H. Sugimoto and O. Shoji, *ChemBioChem*, 2022, **23**, e202200095.
3. N. Izadi-Pruneyre, N. Wolff, V. Redeker, C. Wandersman, M. Delepierre and A. Lecroisey, *Eur. J. Biochem.*, 1999, **261**, 562-568.
4. I. Matsunaga, A. Ueda, T. Sumimoto, K. Ichihara, M. Ayata and H. Ogura, *Arch. Biochem. Biophys.*, 2001, **394**, 45-53.
5. E. T. Yukl, G. Jepkorir, A. Y. Alontaga, L. Pautsch, J. C. Rodriguez, M. Rivera and P. Moenne-Loccoz, *Biochemistry*, 2010, **49**, 6646-6654.
6. M. Gerloch, J. Lewis, F. E. Mabbs and A. Richards, *J. Chem. Soc. A*, 1968, **0**, 112-116.
7. K. Paul, H. Theorell and A. Akeson, *Acta Chem. Scand.*, 1953, **7**, 1284-1287.
8. E. J. Moore, D. Zorine, W. A. Hansen, S. D. Khare and R. Fasan, *Proc Natl Acad Sci U S A*, 2017, **114**, 12472-12477.
9. W. Kabsch, *Acta Crystallogr. Sect. D Biol. Crystallogr.*, 2010, **66**, 125-132.
10. P. Evans, *Acta Crystallogr. Sect. D Biol. Crystallogr.*, 2006, **62**, 72-82.
11. P. R. Evans, *Acta Crystallogr. Sect. D Biol. Crystallogr.*, 2011, **67**, 282-292.
12. P. R. Evans and G. N. Murshudov, *Acta Crystallogr. Sect. D Biol. Crystallogr.*, 2013, **69**, 1204-1214.
13. A. Vagin and A. Teplyakov, *Journal of Applied Crystallography*, 1997, **30**, 1022-1025.
14. A. Vagin and A. Teplyakov, *J. Appl. Crystallogr.*, 1997, **30**, 1022-1025.
15. A. Y. Alontaga, J. C. Rodriguez, E. Schönbrunn, A. Becker, T. Funke, E. T. Yukl, T. Hayashi, J. Stobaugh, P. Moënne-Loccoz and M. Rivera, *Biochemistry*, 2009, **48**, 96-109.
16. P. Emsley and K. Cowtan, *Acta Crystallogr. Sect. D Biol. Crystallogr.*, 2004, **60**, 2126-2132.
17. G. N. Murshudov, P. Skubak, A. A. Lebedev, N. S. Pannu, R. A. Steiner, R. A. Nicholls, M. D. Winn, F. Long and A. A. Vagin, *Acta Crystallogr. Sect. D Biol. Crystallogr.*, 2011, **67**, 355-367.
18. S. Spicher and S. Grimme, *Angew. Chem., Int. Ed.*, 2020, **59**, 15665-15673.
19. C. E. Kibbey and M. E. Meyerhoff, *Anal. Chem.*, 1993, **65**, 2189-2196.
20. R.-S. Lin, M.-R. Li, Y.-H. Liu, S.-M. Peng and S.-T. Liu, *Inorganica. Chimica. Acta.*, 2010, **363**, 3523-3529.
